# Nature is the best source of anticancer drugs: Indexing natural products for their anticancer bioactivity

**DOI:** 10.1371/journal.pone.0187925

**Published:** 2017-11-09

**Authors:** Anwar Rayan, Jamal Raiyn, Mizied Falah

**Affiliations:** 1 Drug Discovery Informatics Lab, QRC - Qasemi Research Center, Al-Qasemi Academic College, Baka EL-Garbiah, Israel; 2 Drug Discovery and Development Laboratory, Institute of Applied Research - The Galilee Society, Shefa-Amr, Israel; 3 Faculty of Medicine in the Galilee, Bar-Ilan University, Ramat Gan, Tel Aviv, Israel; 4 Galilee Medical Center, Nahariya, Israel; Columbia University, UNITED STATES

## Abstract

Cancer is considered one of the primary diseases that cause morbidity and mortality in millions of people worldwide and due to its prevalence, there is undoubtedly an unmet need to discover novel anticancer drugs. However, the traditional process of drug discovery and development is lengthy and expensive, so the application of *in silico* techniques and optimization algorithms in drug discovery projects can provide a solution, saving time and costs. A set of 617 approved anticancer drugs, constituting the active domain, and a set of 2,892 natural products, constituting the inactive domain, were employed to build predictive models and to index natural products for their anticancer bioactivity. Using the iterative stochastic elimination optimization technique, we obtained a highly discriminative and robust model, with an area under the curve of 0.95. Twelve natural products that scored highly as potential anticancer drug candidates are disclosed. Searching the scientific literature revealed that few of those molecules (Neoechinulin, Colchicine, and Piperolactam) have already been experimentally screened for their anticancer activity and found active. The other phytochemicals await evaluation for their anticancerous activity in wet lab.

## Introduction

Cancer is one of the primary global diseases that cause morbidity and mortality in millions of people worldwide [1]. Its incidence is expected to rise by about 70% over the next two decades. Cancer cells can initiate, spread, lodge, and grow in various tissues and organs throughout the body, where the five most common sites of cancer among men are in the lungs, prostate, colorectum, stomach, and liver, and among women in the breast, colorectum, lungs, cervix, and stomach [2]. Current cancer therapies often involve surgical removal and radiation treatment of the large accumulated biomass of cancer, typically followed by systemic chemotherapy treatment used for maintenance treatment. The major disadvantages of chemotherapy are the recurrence of cancer, associated with drug resistance, and severe side effects that can limit the use of anticancer drugs and thus impair patients’ quality of life. Despite this, chemotherapy is still one of the most widely used treatments in all kinds of cancers and at every stage of cancer progression.

The molecular basis of cancer cell development among differentiated normal cells is well studied and has been attributed to two key components, namely oncogenes and tumor suppressor genes [3, 4]. Respective activation and inactivation of these oncogenes and tumor suppressor genes by naturally occurring mutations in either one or both of them can trigger uncontrolled growth and proliferation ending with transformation of cells acquiring carcinogenesis properties [4–7]. Similarly, the inactivation of tumor suppressor genes can result in uncontrolled cell growth [6]. An understanding of the molecular mechanisms underlying cancer progression has led to the development of a vast number of anticancer drugs; however, the use of many chemically synthesized anticancer drugs has caused considerable harm to patients, mainly in the form of immune system suppression. Therefore, the discovery and development of new drugs based on natural products have been the focus of much research [8, 9]. Alkaloids, flavonoids, terpenoids, polysaccharides, saponins and others have been documented as natural bioactive products with potent anticancer activity [10–12]. Most (> 60%) anticancer drugs that are in clinical use and have demonstrated significant efficacy for combatting cancer originate from natural products derived from plants, marine organisms, and microorganisms [13]. The anticancer activity of most natural products often act via regulating immune function, inducing apoptosis or autophagy, or inhibiting cell proliferation.

Nature is the best source of drugs[14, 15] and due to our interest in the identification of new anticancer natural products that overcome the limitations of cell toxicity and adverse reactions, in addition to exhibiting improvements in treatment efficiency, we describe here *in silico* model for indexing natural products for their anticancer bioactivity. The *in silico* studies and mathematical-/statistical-based modeling presented here provide insights into the physicochemical properties associated with anticancer activity at the molecular level. Structural based [16–18] and/or Ligand-based techniques [19, 20] are widely used for constructing predictive models and for the *in silico* screening of large chemical databases, whose aim is to detect novel bioactive ligands [21, 22]. Models for constructing predictive models and separating active from inactive ligands can be developed by selecting sets of active and inactive chemicals for learning purposes and using certain optimization methods (such as neural networks [23], genetic algorithms [24], support vector machines [25], the k-nearest neighbor algorithm, [26, 27], or some combination thereof [27–29]). Modelers presume that chemicals with certain biological properties have common features that are responsible for their bioactivity, but these cannot be easily recognized if an inadequate number of bioactive ligands are tested. To arrive at more significant and robust conclusions, we need to consider large and diverse sets of active/ inactive ligands. As well, the way we select the set of inactive ligands to be used for modeling purposes is highly significant. It should cover the same range of properties possessed by the ligands in the screened database.

The iterative stochastic elimination (ISE) optimization technique is a recent development that has been presented in several research publications[19, 20, 22, 30, 31]. It is an efficient technique for searching a multi-dimensional space in order to identify the best set of solutions (termed *global minima* and *local minima*). ISE has been used to solve problems such as proton positioning in proteins [32], the prediction of side-chain conformations [33], the verification of loop conformations [34], and the conformational space of cyclic peptides [35]. During the last few years, ISE has been applied to solving several chemoinformatics problems [22]; certain sets of physicochemical properties are selected from a large set of physicochemical properties, and the ranges of the selected properties are optimized to produce the best set of solutions (termed *filters*) capable of separating active from inactive ligands. The constructed filters are jointly applied to index ligands for their bioactivity and to rank and prioritize molecules in large chemical databases [20, 30, 36].

In this paper, we disclose a novel model for indexing natural products for their potential anticancer activity, and map the discriminative physicochemical properties of 617 FDA-approved anticancer drugs through careful analysis of the composition of filters that were produced by ISE for indexing purposes.

## Methods

**Fig 1:**
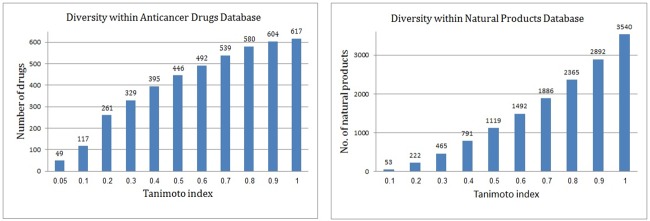
Diversity within anticancer drugs (A, left side) and diversity within natural products database (B, right side).

To construct the predictive model, we used a set of 617 anticancer drugs to constitute the active domain (all anticancer drugs are presented in SMILES format followed by their common names in the supporting information S1 Table). This set of drugs was assembled from CMC (Comprehensive Medicinal Chemistry) database and NCI Drug Dictionary. Another set of 2,892 natural products was used to constitute the inactive domain. This database of natural products was prepared by collecting phytochemicals that were isolated from more than eight hundred diverse plants spread worldwide and are deliverable from AnalytiCon Discovery (www.ac-discovery.com). To obtain the data set of natural products, go to the link https://ac-discovery.com/downloads/ and download "Purified Natural Products". At the first time, each new user need to register and then sign in for file download. We believed that a very small fraction of the 2,892 natural products that were assigned as inactive were actually active ligands. However, from our experience in previous projects, such assignment was justified and beneficial, since (1) the model used for virtual screening should cover the same range of properties as those possessed by the chemicals in the screened database (the natural products database used herein was prepared by collecting phytochemicals isolated from plants, and (2) the effect on model quality is minor if the portion of really false negatives in the training set is less than 1–2%. The Tanimoto index-based diversities within both databases (anticancer drugs and natural products) are shown in Fig 1.

The physicochemical properties (descriptors) of all the ligands in both databases (the active/anticancer drug and inactive/natural product DBs) were calculated using Molecular Operating Environment (MOE) software, version 2009.10, [http://www.chemcomp.com]. The calculated 1-dimensional (1D)/2-dimensional (2D) descriptors were of physicochemical properties such as molecular weight, log P, H-bond acceptors/donors, solubility, total charge and charge distribution, the types and numbers of atoms, etc. (http://www.chemcomp.com/journal/descr.htm). An assessment of the constructed models and validation of their predictability was done by splitting the datasets of the active/inactive ligands into 66.7% for training and 33.3% for testing. Both training and test sets were generated by an in-house random picking module.

**Fig 2:**
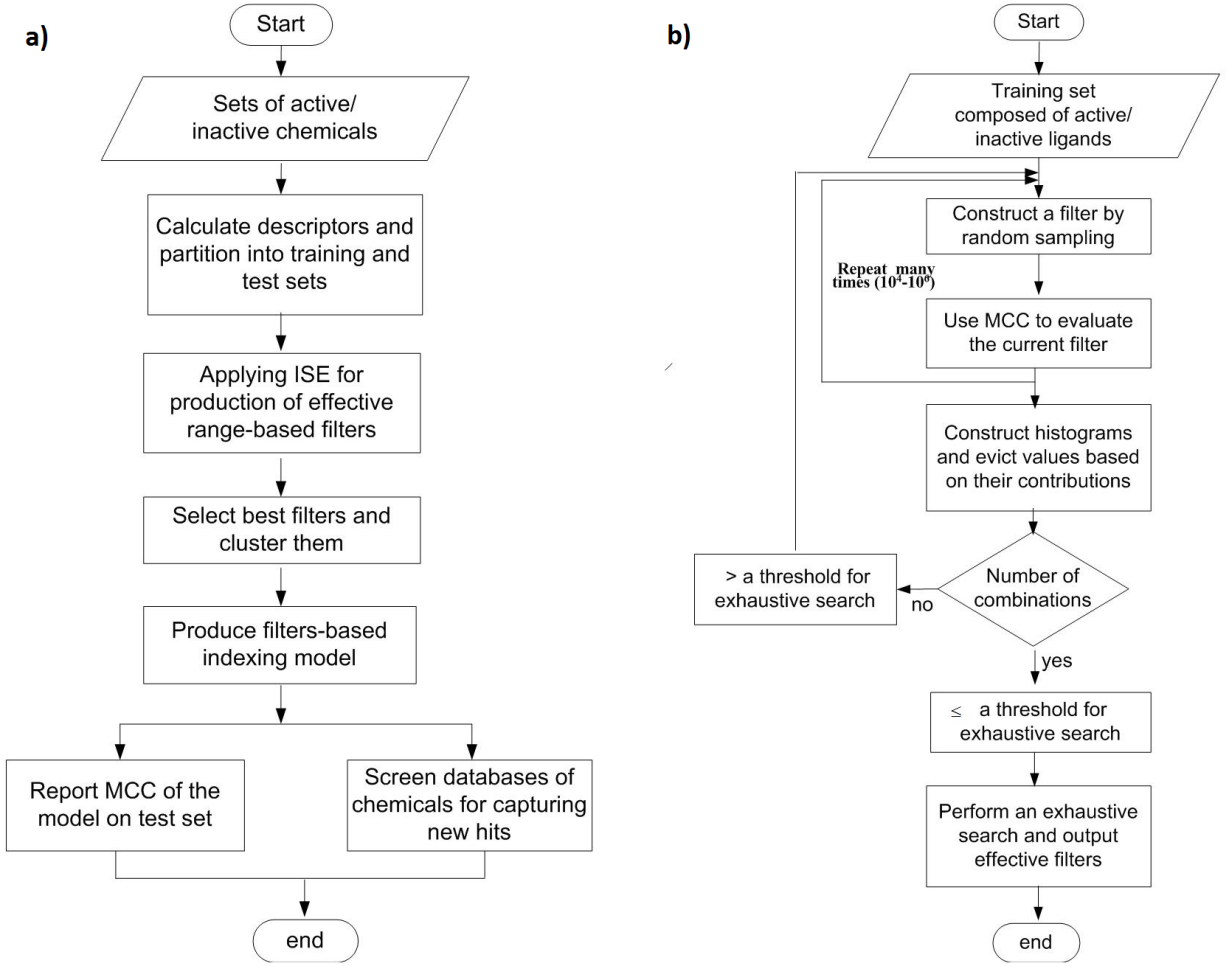
Flowcharts for the modeling process (2a), and the ISE engine (2b).

The ISE algorithm was utilized to build a prediction model capable of indexing natural products for potential anticancer activity. According to our algorithm [20], the optimal model capable of differentiating between active and inactive ligands was obtained by searching multivariable space for the best sets of descriptors (termed *variables*) and the best range of each descriptor that separated the active from inactive ligands. The optimization process was highly complicated, since the physicochemical properties of the ligands interact with each other, and changes in the range of one property may affect the best range of other properties that compose the same filter. The optimization process must consider all of the properties of the filter at the same time. Fig 2 summarizes the main points of the ISE-based modeling process. More details on the utility of ISE for extracting the best sets of descriptors, as well as the best ranges, from a certain set of descriptors can be found in our previously reported studies [20, 30].

## Results and discussion

**Fig 3:**
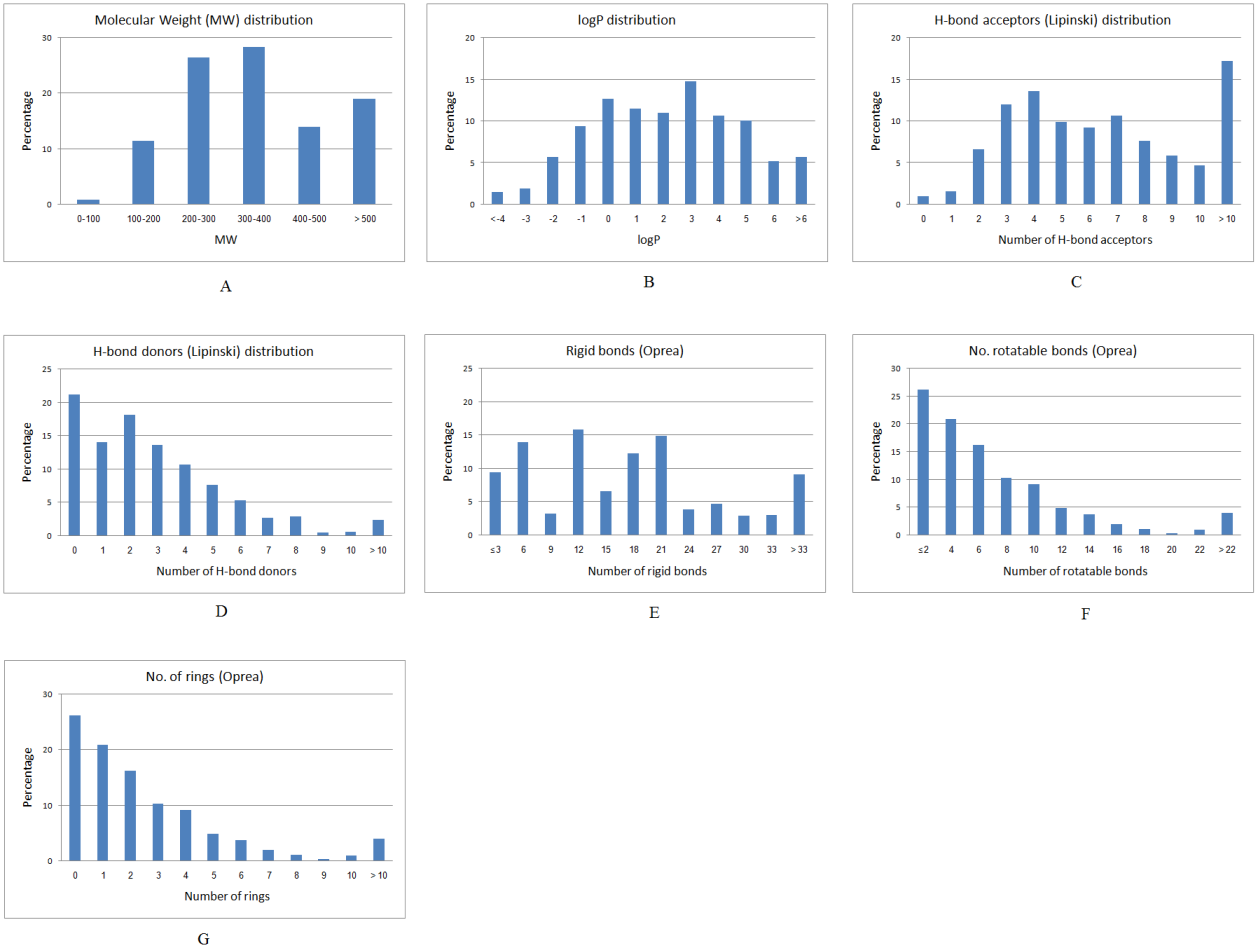
Physicochemical properties distribution of anticancer drugs (A) Molecular weight distribution, (B) Log P values, (C) Number of H-bond acceptors [lip_acc], (D) Number of H-bond donors [lip_don], (E) Number of rigid bonds, (F) number of rotatable bonds, (G) Number of aromatic atoms.

**Fig 4:**
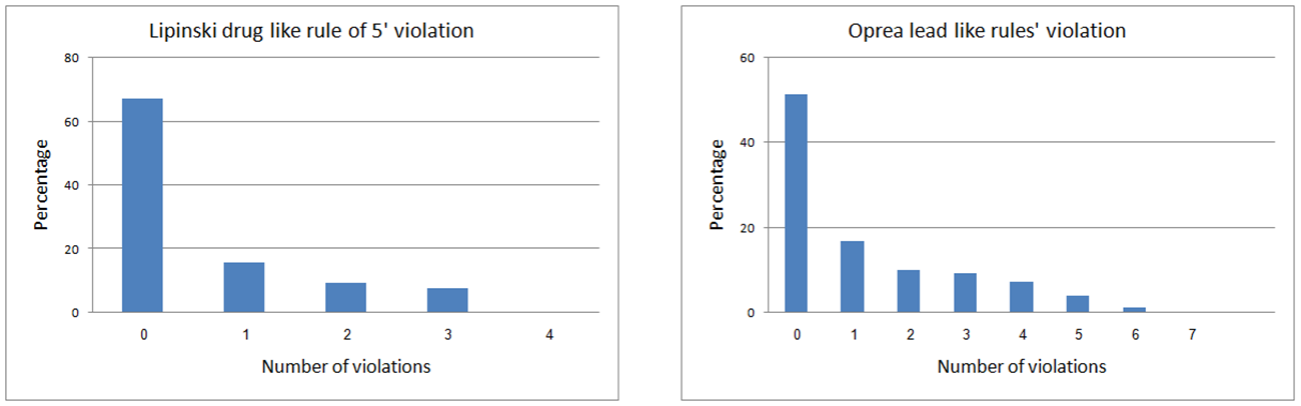
Violation distribution of anticancer drugs to Lipinski rule of 5 for drug-likeness (left side) and Oprea rule for lead-likeness (right side).

The ISE algorithm was applied to construct an *in silico* prediction system for detecting natural products with potential anticancer activity. This study was based on a set of 617 anticancer drugs labeled as active chemicals and 2,892 natural products labeled as inactive phytochemicals. It is worth noting that a few of the 2,892 natural products had the potential to be anticancer compounds, but the effect of that assumption on the quality of the prediction model was negligible, especially since the fraction of active products was expected to be less than 1–2% (data not shown). From previous projects, we learned that predictive models for virtual screening purposes should cover the same range of properties as those possessed by the objects in the screened database. In light of that, we selected, as the inactive set, chemicals with the same "property range" as the chemicals in the screened database. As well, in order to make sure that our active set of chemicals would not be biased by having similar structures, we checked the structural diversity within the 617 anticancer drugs and the 2,892 natural products and found that both databases were highly diverse. 86 of the anticancer drugs and 53 of the natural products had a Tanimoto index of similarity < 0.7. As shown in Fig 3, it is interesting to note that 83% of the anticancer drugs obeyed Lipinski’s Rule of Five (ROF), and 68% obeyed the Oprea rules for lead-likeness [37]. Fig 4 presents distribution plots of the Lipinski and Oprea physicochemical properties of the set of anticancer drugs.

The indexing model was produced by 29 unique filters, which consisted of different sets of descriptors and/or same set of descriptors with different ranges. Table 1 presents three of the filters as an example. The Matthews correlation coefficients (MCCs) of the different filters are very close, but they differ in their true positive percentage and true negative percentage. Filter number 1, presented in Table 1, has a MCC of 0.568, and with this filter, 53.7% of the anticancer drugs were successfully identified as true positives, while only ~2.5% of the natural products database were classified as active. The filter is composed of ranges of four descriptors. Each molecule that fall within these ranges is considered active; while molecules having as least one descriptor that fall outside the range is considered inactive. It is worth stating that we presumed that most of the screened natural products were inactive, and thus, this classification is considered a false positive, although we are aware that some of those natural products were active and were correctly classified by our proposed prediction model.

**Table 1:** Three filters out of the 29 filters used for producing the anticancer indexing model. The Matthews correlation coefficients (MCCs), the true positive (TP) percentages, the true negative (TN) percentages, and the descriptors' ranges are shown.

*Filter 1*	*Filter 2*	*Filter 3*
*MCC = 0*.*568*	*MCC = 0*.*554*	*MCC = 0*.*551*
*TP = 53*.*64%*	*TP = 63*.*37%*	*TP = 50*.*57%*
*TN = 97*.*48%*	*TN = 90*.*04%*	*TN = 97*.*99%*
*GCUT_SLOGP_0 (-2*.*28−-0*.*89)*	*a_ICM (1*.*47–2*.*21)*	*chiral_u (0*.*– 2*.*0)*
*BCUT_SLOGP_3 (-0002*. *- 2*.*97)*	*PEOE_VSA-5 (0*. *- 393*.*44)*	*b_rotR (0*. *- 0*.*80)*
*vsa_don (0*.*24–247*.*82)*	*PEOE_VSA_FPNEG (0*. *- 0*.*516)*	*vsa_don (0*.*24–247*.*82)*
*PEOE_VSA+0 (0*. *- 672*.*01)*	*PEOE_VSA+4 (0*. *- 51*.*58)*	*b_1rotR (0*. *- 0*.*80)*

**Fig 5:**
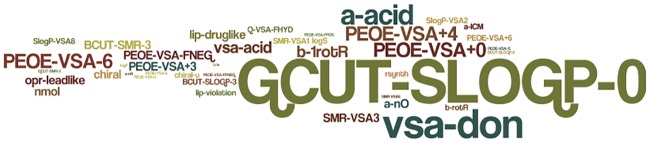
Redundancy of descriptors in the 29 filters used to produce the anticancer indexing model. The picture was constructed by using WORDLE module.

The composition of the output list of best discriminative filters was analyzed. Table 2 lists the most redundant descriptors of the 29 filters used to produce the anticancer indexing model. The third column reports how many more times each descriptor was redundant rather than random. Fig 5 was built using the WORDLE module; it displays the redundancy of the descriptors in graphical mode.

**Table 2:** Descriptors' redundancy.

Descriptor name	Redundancy	Redundant more times than random
GCUT_SLOGP_0	15	24.1
a_ICM	10	16.0
PEOE_VSA+4	7	11.2
SMR_VSA1	5	8.0
logS	5	8.0
Nmol	5	8.0
lip_druglike	4	6.4
chi1_C	4	6.4

**Fig 6:**
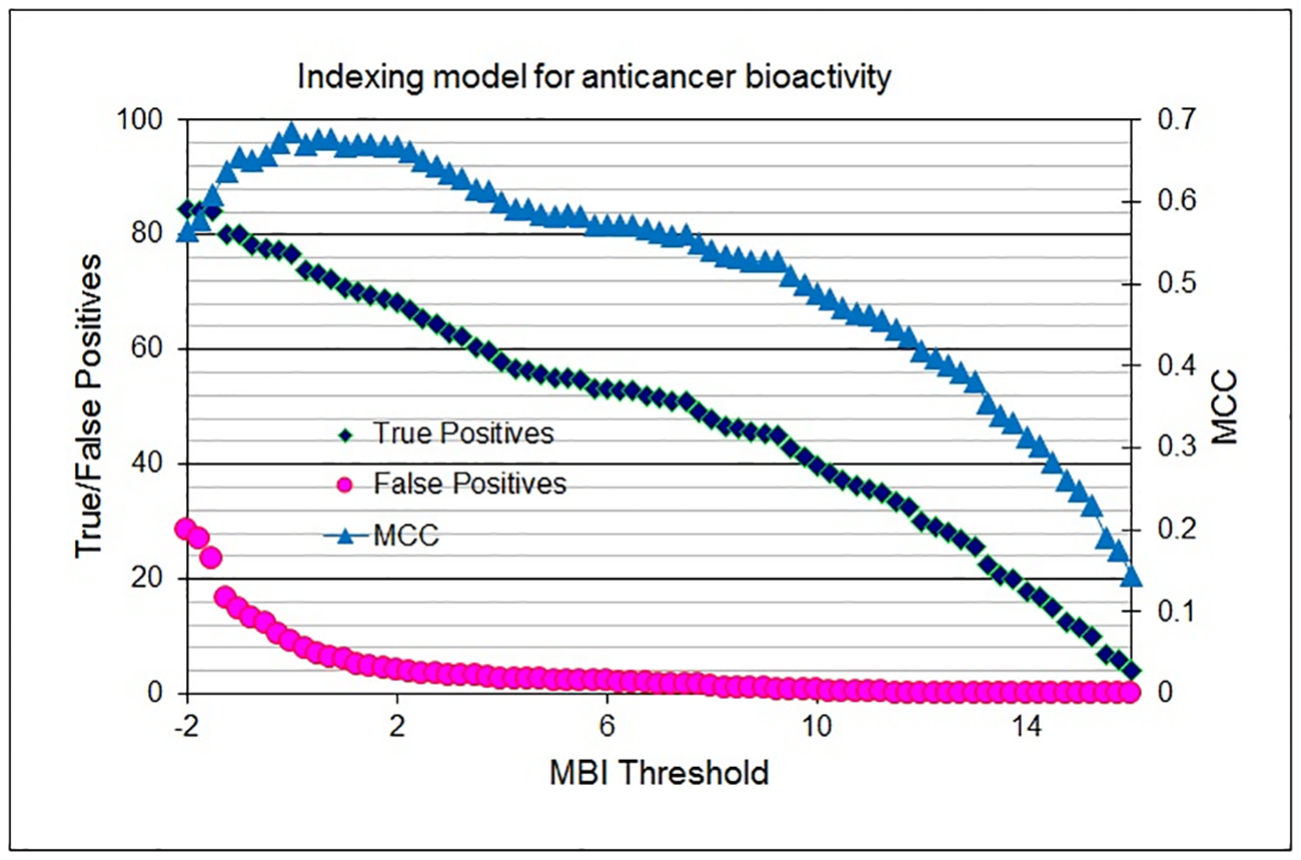
Indexing model for anticancer potential activity: True/false positives percentage (left Y-axis) and Matthews's correlation coefficient (MCC, right Y-axis) illustrated against molecular bioactivity index threshold (MBI, X-axis).

The efficiency of the anticancer activity-indexing model, which was produced by the 29 range-based filters, is displayed in Fig 6. The true/false positive percentage (left *y*-axis) and Matthews's correlation coefficients (right *y*-axis) are plotted against the molecular bioactivity index thresholds (*x*-axis).

**Fig 7:**
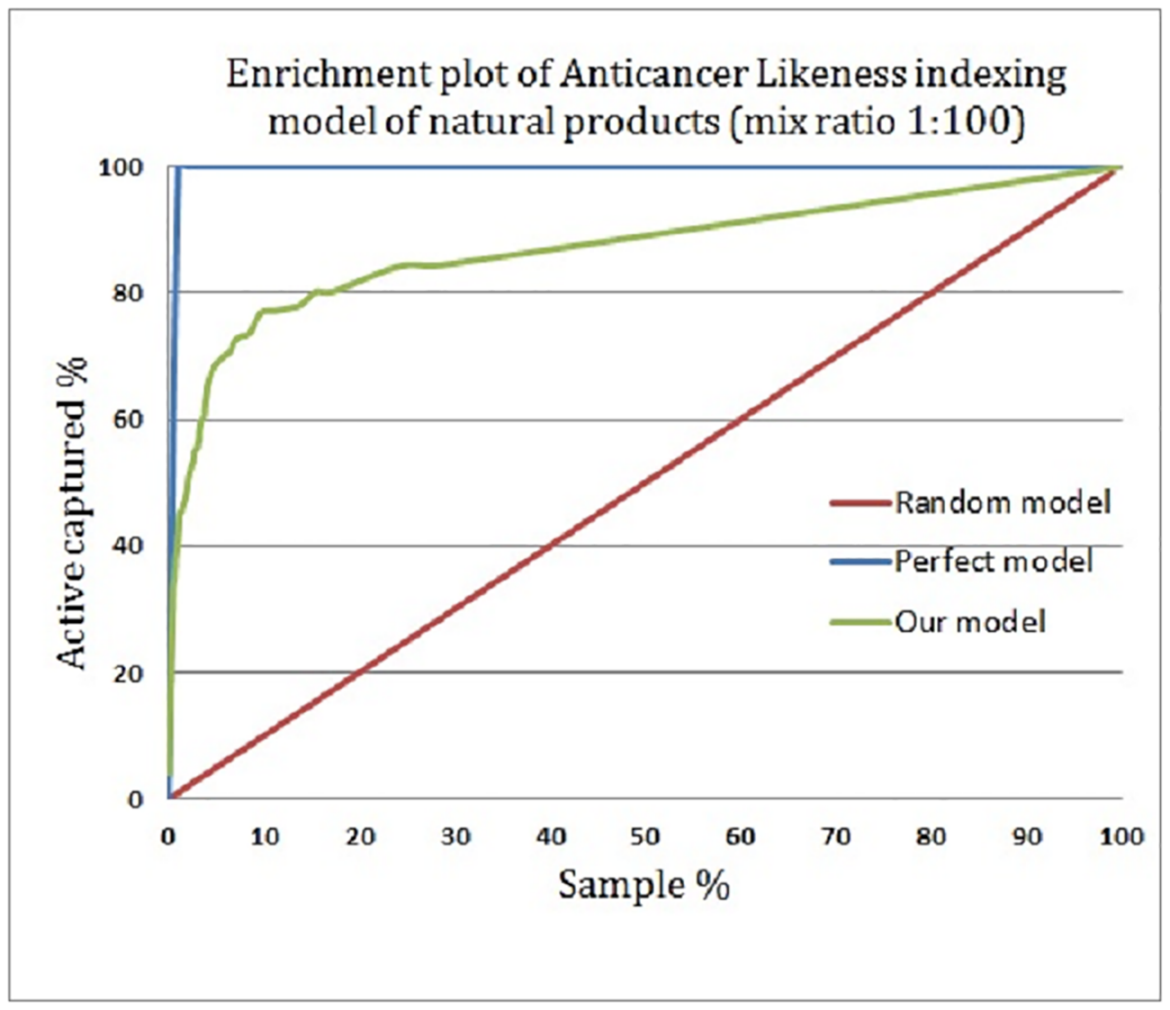
Enrichment plot of the anticancer potential activity-indexing model of natural products.

**Fig 8:**
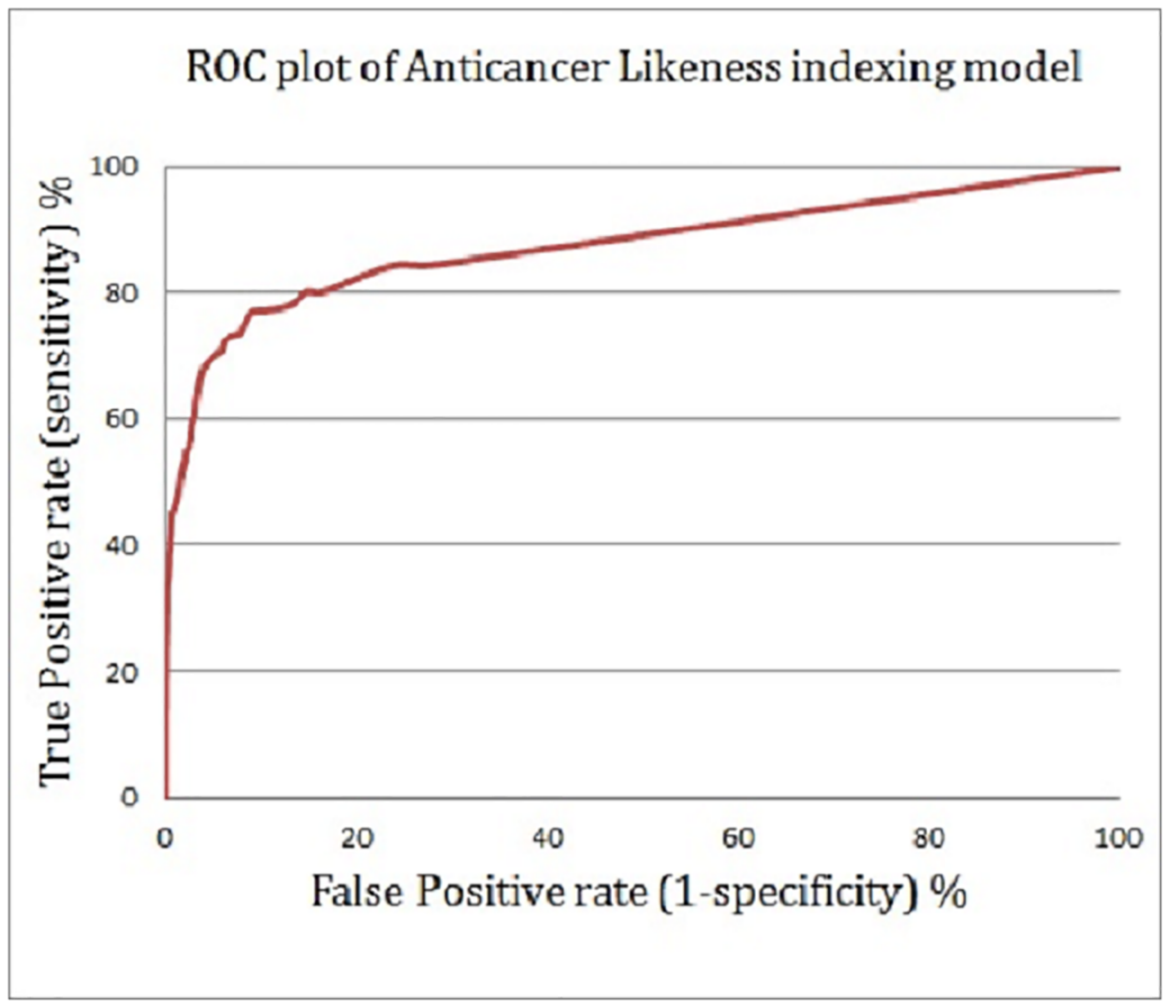
A receiver operating characteristic (ROC) curve showing the performance of the anticancer bioactivity-indexing model.

Figs 7 and 8 show the enrichment plot and the receiver operating characteristic (ROC) plot of the suggested anticancer bioactivity-indexing model, respectively. The enrichment plot (Fig 7) illustrates how the anticancer drug candidates could be predicted if natural products are ranked according to their scores as predicted by the ISE-based model, rather than based on random selection. An enrichment plot where the ISE-based model overlaid with the perfect model at the one percent highest fraction indicates the high prioritization power of the constructed model. By applying this proposed anticancer bioactivity indexing model at a mix ratio of 1:100 (active/ inactive), 42% of the anticancer drugs could be captured in the top one percent of the screened compounds, compared with 100% in the perfect model and 1% in the random model.

**Fig 9:**
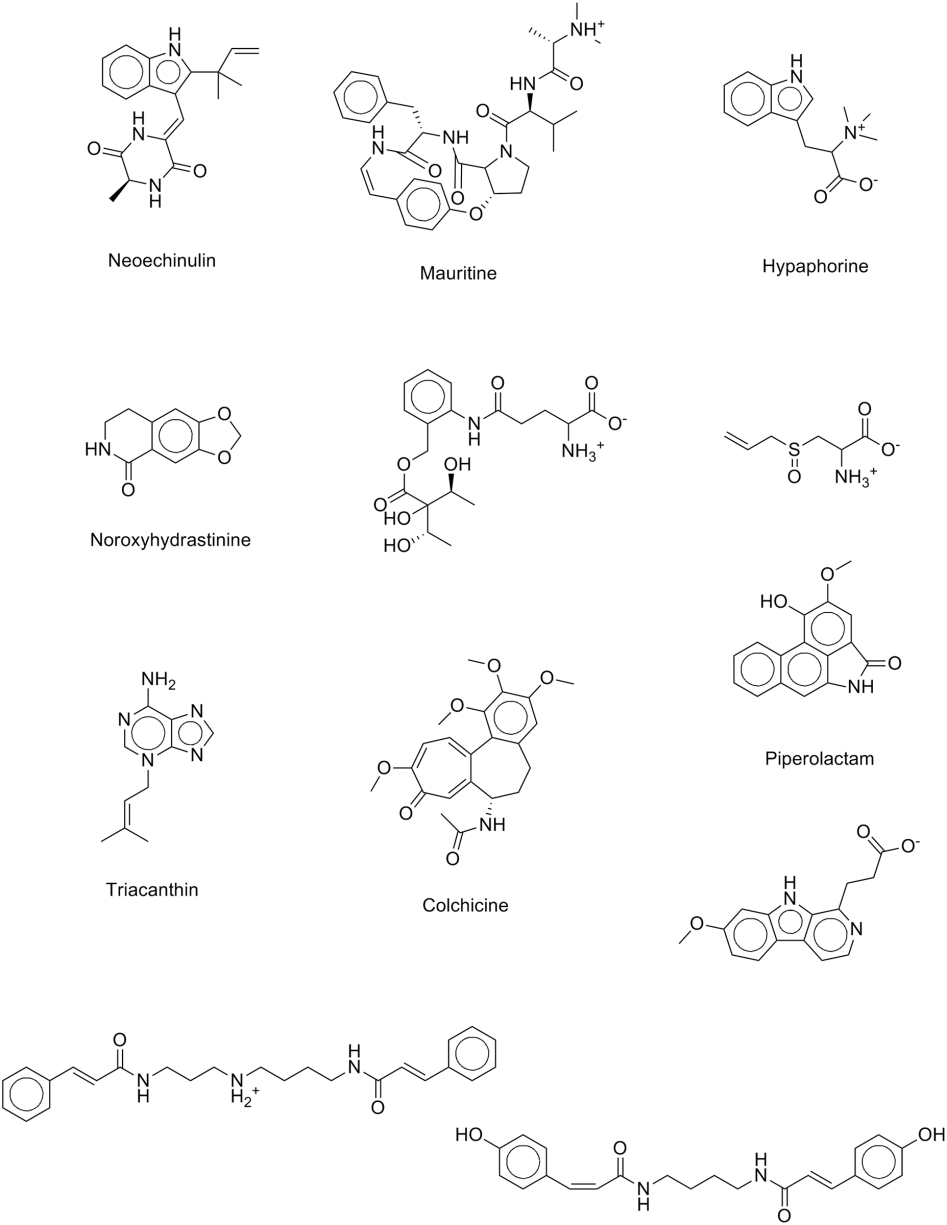
Twelve of the natural products that are scored highly as potential anticancer drug candidates according to our ISE-based anticancer indexing model.

The attained area under the curve (AUC) of the proposed ISE-based model is 0.95, indicating the effectiveness of the model. As well, the ISE-based model and the perfect model overlap somewhere in the range of molecular bioactivity index (MBI) ≥ 4.0; thus, the model is considered highly discriminative and effective for classifying anticancer drug candidates and inactive natural products. Fig 9 shows twelve natural products that were highly indexed as potential anticancer drug candidates by our ISE-based anticancer indexing model. Searching the scientific literature revealed that few of those molecules (Neoechinulin[38**]**, Colchicine[39], and Piperolactam[40]) have already been experimentally screened for their anticancer activity and found active. The other phytochemicals await evaluation for their anticancerous activity in wet lab.

## Conclusions

A highly efficient and robust model for indexing natural products for their anticancer bioactivity has been built using the ISE algorithm. We believe that the use of such an *in silico* model to screen large databases of natural products could undoubtedly save time and costs and aid in detecting novel natural-based anticancer drug candidates. We have disclosed some highly indexed phytochemicals that could serve as potential anticancer drug candidates. A literature search shows that few of those molecules have already been experimentally screened for their anti-cancerous activity and found active. The other phytochemicals await evaluation for their anti-cancerous activity in wet lab. As well, this study provides important insights into discriminative properties of natural products having anti-cancerous activity.

## Supporting information

S1 Table 617 anticancer drugs are presented below in SMILES format followed by their common names. (PDF)

## Abbreviations

EF: enrichment factor
FP: false positive
ISE: iterative stochastic elimination
MBI: molecular bioactivity index
MCC: Matthews’ correlation coefficient
MD: molecular dynamics
TP: true positive
vHTS: virtual high-throughput screening
