# Supplementary material for: Nature is the best source of anticancer drugs: Indexing natural products for their anticancer bioactivity
Source: PLoS One. 2017 Nov 9;12(11):e0187925. doi: 10.1371/journal.pone.0187925 (PMC5679595; doi:10.1371/journal.pone.0187925)
Supplement: S1 Table — (PDF) [file pone.0187925.s001.pdf]

Supporting information

S1 Table: 617 anticancer drugs are presented below in SMILES format followed by their common names

O([C@]12[N](C=3C(C(C)=C(C(C3[C@@H]2COC(N)=O)=O)N)=O)C[C@@H]2[C@@H]1N2)C MITOMYCIN

c12c(nc[nH]c1=S)nc[nH]2 MERCAPTOPURINE

c1([N]2CC2)nc([N]2CC2)nc(n1)[N]1CC1 TRIETHYLENEMELAMINE

c1(c(c[nH]c([nH]1)=O)F)=O FLUOROURACIL

C([N](CCCl)C)CCl MECHLORETHAMINE

O(C(N)=O)CC URETHANE

S=[P]([N]1CC1)([N]1CC1)[N]1CC1 THIOTEPA

C([C]1(CCCC1)N)(O)=O CYCLOLEUCINE

c1([CH](c2ccc(Cl)cc2)[CH](Cl)Cl)c(cccc1)Cl MITOTANE

O1[C@@H]([n@@]2c([nH]c(=O)cn2)=O)[C@@H]([C@@H](O)[C@H]1CO)O AZAURIDINE

C([N]1CC[N](C(CCBr)=O)CC1)(CCBr)=O PIPOBROMAN

O([S@@](C)(=O)=O)CCCCO[S@@](C)(=O)=O BUSULFAN

c12[C@]34[C@@H]5([C]([C@@H](OC(C)=O)[C@@]([C@@H]3([N](C=O)c1cc(OC)c(c2)[C]1(c2c(c3ccccc3[nH]2)CC[N]2C[C@@H](C1)(C[C@](CC)(O)C2))C(OC)=O))(C(OC)=O)O)(C=CC[N]5CC4)CC) VINCRISTINE

C1[C@@H]2([C@@H]([C]3([C@@H](CC(=O)[C@@H](C3)C)(C1))C)(CC[C]1([C@H]2(CC[C@@H]1O))C)) DROMOSTANOLONE

O1[C@@H]([n@]2c([nH]c(=O)c(c2)Br)=O)(C[C@@H](O)([C@H]1(CO))) BROXURIDINE

c1(c(c(c(C)nc1)O)C)CO DESOXYPYRIDOXINE

O([S](C)(=O)=O)C METHYL METHANESULFONATE

c1([N](CCCl)CCCl)c([nH]c(=O)[nH]c1)=O URACIL MUSTARD

C1(=C([N]2CC2)C(C=C(C1=O)[N]1CC1)=O)[N]1CC1 TRIAZIQUONE

c12[n@@]([C@@H]3O[C@H](CO)[C@H]([C@H]3O)O)ccc1c(ncn2)N TUBERCIDINE

c1(C=2NCCN2)ccc(NC(c2cc(cc(C(Nc3ccc(C=4NCCN4)cc3)=O)cc2)Cl)=O)cc1 NSC 60339

c1([C@H]([C@H](c2ccc(O)cc2)CC)CC)ccc(O)cc1 MESO-HEXESTROL

c12c(C(C(O)=C(C1=O)C\C=C(\C)C)=O)cccc2 LAPACHOL

C(C(C)=C)(OC[CH]1CO1)=O GLYCIDYL METHACRYLATE

C(NC)=O METHYLFORMAMIDE

O=[P]([N](c1nncs1)CC)([N]1CC1)[N]1CC1 AZATEPA

c1([C]2(C(NC(=O)CC2)=O)CC)ccc(N)cc1 AMINOGLUTETHIMIDE

C(NO)(N)=O HYDROXYUREA

c12c(c([nH]c(n1)N)=O)[nH]nn2 AZAGUANIDINE

c12[n@@]([C@@H]3O[C@H](CO)[C@H]([C@@H]3O)O)cnc1c(nc(n2)F)N FLUOROADENOSINE

c12c(C[CH](C(O)=O)N)c[nH]c1ccc(c2)[N](CCCl)CCCl TRYPTOPHANE MUSTARD

c12c(c([nH]c(n1)N)=S)[nH]cn2 THIOGUANINE

C([N](CCCl)N=O)(NCCCl)=O CARMUSTINE

O([S](C)(=O)=O)C[C@@H]([C@H](CO[S](C)(=O)=O)O)O TREOSULFAN

N([P]([N]1CC1)([N]1CC1)=O)C(OCC)=O UREDEPA

c1([N](CCCl)CCCl)ccc(CCCC(O)=O)cc1 CHLORAMBUCIL

O1[C@@H]([n@]2c(nc(N)nc2)=O)[C@@H]([C@@H](O)[C@H]1CO)O AZACITIDINE

c12[n@@]([C@@H]3O[C@H](CO)[C@H]([C@H]3O)O)cnc1c(ncn2)SC METHYLTHIOINOSINE

c1(c(C[CH](C(O)=O)N)cccc1)[N](CCCl)CCCl MEROPHAN

C1[C@H]2([C@H]3([C]([C@](C(CF)=O)(O)CC3)(C[C@@H]([C@@H]2([C]2(C(=CC(=O)CC2)[C@@H]1C)C))O)C)) NSC 19622

C1[C@@H]2([C@@H]([C]3(C(=CC(=O)CC3)[C@H]1F)C)(CC[C]1([C@H]2(CC[C@@H]1OC(CC)=O))C)) FLUOROTESTOSTERONE

C1[C@@H]2([C@@H]([C]3(C(=CC(=O)CC3)[C@@H]1F)C)(CC[C]1([C@H]2(CC[C@@H]1OC(CC)=O))C))6-FLUOROTESTOSTERONE PROPIONATE

C1[C@H]2([C@H]3([C@@H]([C]4(C(=CC(=O)CC4)CC3)C)([C@@H](O)C[C]2([C@](C(CF)=O)(O)C1)C))) DIHYDROXYFLUOROPROGESTERONE

C1(=C(C(C([N]2CC2)=C(C1=O)OCCC)=O)OCCC)[N]1CC1 INPROQUONE

C1[C@@H]2([C@@H]([C]3([C@@H](C[C@@H](F)CC3)(C1))C)(CC[C]1([C@H]2(CC[C@@H]1O))C)) 3-FLUOROANDROSTANOL

C(=N/NC(N)=N)(/C=N/NC(N)=N)C MITOGUAZONE

O[C@@H]([C@@H]([C@@H](CBr)O)O)[C@@H](CBr)O MITOBRONITOL

c1([N](CCCl)CCCl)cc2c(cccc2)cc1 CHLORNAPHAZINE

C1[C@@H]2([C@@H]([C]3([CH](CC(=O)[C@@H](C3)(C))C1)C)(CC[C]1([C@H]2(CC[C@@H]1(OC(CC)=O)))C)) DROMOSTANOLONE PROPIONATE

c12c(ncc(n1)C[N](c1c(cc(C(N[C@@H](CCC(O)=O)C(O)=O)=O)cc1Cl)Cl)C)nc(N)nc2N DICHLOROMETHOTREXATE

c1([N](CCCl)CCCl)cc(ccc1)C[C@@H](C(O)=O)N SARCOLYSINE

O=[P@]([N]1CC1)([N]1CC1)[N]1CC1 TRIETHYLENEPHOSPHORAMIDE

C(=C(\c1ccc(O)cc1)C)(/c1ccc(O)cc1)C DIMETHYLSTILBESTROL

c1([N](CCCl)CCCl)ccccc1 ANILINE MUSTARD

C([N](CCCl)CCCl)CCl TRICHLORMETHINE

c12[n@@]([C@@H]3O[C@H](CO)[C@H]([C@H]3O)O)cnc1c([nH]cn2)=S THIOINOSINE

O[CH]([CH]([CH](CNCCCl)O)O)[CH](CNCCCl)O MANNOMUSTINE

c1(c2c(c3ccc(cc3[n+]1CC)N)ccc(c2)N)c1ccccc1.[Cl-] ETHIDIUM CHLORIDE

c12[n@@]([C@@H]3O[C@H](CO)[C@H]([C@H]3O)O)cc(C#N)c1c(ncn2)N TOYOCAMYCIN

O1[C@@H]2[C@H]([C@H](OC(C)=O)C1=O)OC([C@H]2OC(C)=O)=O ACEGLATONE

c1(nc(nc(n1)[N](C)C)[N](C)C)[N](C)C ALTRETAMINE

c1(C(N[CH](C)C)=O)ccc(CNNC)cc1 PROCARBAZINE

c12[n@@]([C@@H]3O[C@H](CO)[C@H](C3)O)cnc1c(nc([nH]2)N)=S THIOGUANINE BETA-DEOXYRIBOSIDE

O([C@@]12[N](C=3C(C(C)=C(C(C3[C@H]2(COC(N)=O))=O)N)=O)C[C@H]2[C@H]1[N@@]2C)C PORFIROMYCIN

N(CC(NN)=O)C(C=[N+]=[N-])=O DIAZOACETYLGLYCINE HYDRAZIDE

c1([N](CCF)CCCl)c([nH]c(=O)[nH]c1=O)C FLUORODOPANE

c12[C@]34[C@@H]5([C]([C@@H](OC(C)=O)[C]([C@@H]3([N](C)c1cc(OC)c(c2)[C]1(c2c(c3ccccc3[nH]2)CC[N@]2C[C@@H](C1)(C[C](CC)(O)C2))C(OC)=O))(C(OC)=O)O)(C=CC[N]5CC4)CC) VINBLASTINE

c12[C@]34[C@@H]5([C]([C@@H](OC(C[N](C)C)=O)([C@@]([C@@H]3([N](C)c1cc(OC)c(c2)[C@@]1(c2c(c3ccccc3[nH]2)CC[N]2C[C@@H](C1)C[C@](CC)(O)C2)C(OC)=O))(C(OC)=O)O))(C=CC[N]5CC4)CC) VINGLYCINATE

c12[n@@]([C@@H]3O[C@H](CO)[C@@H](C3)O)cnc1c([nH]cn2)=S ARABINOSYLMERCAPTOPURINE

C([n]1cccn1)(N)=O CARZOLAMIDE

C1[C@@H]2([C]3(C(CC[C@H]2([C@H]2([C](OC(=O)CC2)(C)C1)))=CC(=O)C=C3)C) TESTOLACTONE

C1[C@@H]2([C@@H]([C]3([C@@H](CC(=O)[C@@H](C3)C)(C1))C)(CC[C]1([C@H]2(CC[C@@H]1O[CH]1CCCCO1))C)) NSC 81408

c12c(nc3ccccc3[n+]1C)c(cc(c2)N)N.[Cl-] DIAMINOMETHYLPHENAZINIUM CL

C1[C@H]2([C@H]([C@H]3([C]([C@@H](O)CC3)(C)C1))(CCC=1[C@@H]2(C[C@@H](C)C(C1)=O))) 2-METHYL-19-NORTESTOSTERONE

C1[C@H]2([C@H]3([C](CC[C@@H]2([C@@H]2(C(=CC(=O)[C@@H](C2)C)C1)))([C@@](C)(O)CC3)C)) DIMETHYLNORTESTOSTERONE

C1[C@@H]2([C@@H]([C]3(C(=CC(C3)=O)C1)C)(CC[C]1([C@H]2(CC[C@@H]1C(C)=O))C)) A-NORPROGESTERONE

C1[C@H]2([C@H]3([C@@H]([C]4(C(=CC(=O)CC4)CC3)O)(CC[C]2([C@@](C#C)(O)C1)C))) 10-HYDROXYNORETHISTERONE

C1[C@@H]2([C]3([C@@H](CC[C@H]2([C@H]2([C]([C@@H](OC(C)=O)CC2)(C)C1)))(CC=C(C3)C#N))C) NSC 82484

c1(nc(N)[nH]n1)N GUANAZOLE

c12[C@@H]([C@@H]([C@@H](CO)[C@@H](c1cc1OCOc1c2)O)C(NNCC)=O)c1cc(c(OC)c(c1)OC)OC MITOPODOZIDE

C([N]([P](N)(O)=O)CCCl)CCl PHOSPHORAMIDE MUSTARD

C1[C@@H]2([C@@H]([C]3([C@@H](CC(=O)[C@@H](C3)F)(C1))C)(CC[C]1([C@H]2(CC[C@@H]1O))C)) FLUOROHYDROXYANDROSTERONE

N([P@@]([N]1[C](C)(C)C1)([N]1[C](C)(C)C1)=O)C(OCC)=O METUREDEPA

C1(c2ccc(OC)cc2)=C([CH]([CH](C(O)=O)CC1)C)CC CARBESTROL

C([n]1cccn1)(N)=S THIOCARZOLAMIDE

C=1(c2c(CCC1c1ccccc1)cc(OC)cc2)c1ccc(OCC[N]2CCCC2)cc1 NAFOXIDINE

C1[CH](O1)COCCOCCOCCOC[CH]1CO1 ETOGLUCID

C=12[C@H]3([C@@H]([C]4(C(=CC(=O)CC4)CC3)C)(CCC1[C](C)(C)CC2)) DIMETHYLNORANDROSTADIENONE

N([P]([N]1CC1)([N]1CC1)=O)C(OCc1ccccc1)=O BENZODEPA

c1(NC(c2ccc(C(Nc3ccc(C(NC)=N)cc3)=N)cc2)=N)ccc(C(NC)=N)cc1 AMIDINO TIC

c12[n@]([C@H]3O[C@H](CO)[C@H](C3)O)cnc1c(nc([nH]2)N)=S THIOGUANINE ALPHA-DEOXYRIBOSIDE

c12c(cc3ccc(cc3[o+]1)[N](CC)CC)ccc(c2)[N](CC)CC.[Cl-] PYRONINE B

C1[C@]2([C@]3([C]4([C@@H](C=C(C)CC4)(O[C@@H]2([C@@H](O)[C@H]3OC(C)=O)))COC(C)=O)C)O1 ANGUIDINE

C1[C]2([C@@H]3([C@H]([C@H]4([C@]([C@@H](O)CC4)(C)CC3))(CC[C@H]2(C[C@H]2([C@H]1(S2))))))C EPITIOSTANOL

C1(C(NC(=O)N=C1)=O)=[N+]=[N-] DIAZOURACIL

C1([C]2([C@H]([C@H]3([C](OC(=O)CC3)(C)C1))(CCC=1[C]2(CCC(C1)=O)C))F)=O 9-FLUOROXOTESTOLOLACTONE

C1[N](CC[N](C1)C(CCO[S](C)(=O)=O)=O)C(CCO[S](C)(=O)=O)=O PIPOSULFAN

C1[C@@H]2([C@@H]([C]3(C(=CC(=O)[C@@H](C3)C)C1)C)(C(C[C]1([C@H]2(CC[C@@H]1C(C)=O))C)=O)) 2-METHYL-11-OXOPROGESTERONE

c1([C](c2ccccc2)(c2ccccc2)SC[C@@H](C(O)=O)N)ccccc1 TRITYL CYSTEINE

C1[C@@H]2([C@@H](c3c(cc(OC([N](CCCl)CCCl)=O)cc3)C1)(CC[C]1([C@H]2(CC[C@@H]1(O)))C)) ESTRAMUSTINE

C1[C@@H]2([C@@H]([C]3(C(C[C@@H](OCC[N](CC)CC)CC3)=C1)C)(CC[C]1([C@H]2(CCC1=O))C)) NSC 70801

C(NCCCCCCNC(C)=O)(C)=O HMBA

O1[C@@H]([n@]2c([nH]c(=O)c(c2)F)=O)[C@@H]([C@@H](O)[C@H]1C)O DOXIFLURIDINE

C1[C@@H]2([C]([C@H](C(C)=O)[C@@H]1SC)(CC[C@H]1([C@H]2(CCC=2[C]1(CCC(C2)=O)C)))C) 16-METHYLTHIOPROGESTERONE

C1[C@@H]2([C@@H]([C]3([C@H](CC(=O)CC3)(C1))C)(CC[C]1([C@H]2(CC[C@]1(C)O))C)) HYDROXYMETHYLANDROSTANONE

C([N@]([C@H](C(O)=O)C)CCCl)CCl ALANINE MUSTARD

c1(c2c(c(c(OC)cc2)OC)O)c(c(c2nc3c(C(C(OC)=C(C3=O)N)=O)cc2)nc(c1C)C(OC)=O)N METHYLSTREPTONIGRIN

C1[C@@H]2([C@@H]([C]3(C(C[C@@H](OC(Cc4ccc([N](CCCl)CCCl)cc4)=O)CC3)=C1)C)(CC[C]1([C@H]2(CC[C@@H]1[C@@H](CCC[CH](C)C)C))C)) PHENESTERIN

O([S](C)(=O)=O)CCCl CHLOROETHYL MESYLATE

c1([N](CCCl)CCCl)ccc(C[CH](C(N[CH](C(OCC)=O)C[CH](C)C)=O)NC(C)=O)cc1 ASALEY

C1([C@H]2([C@H]([C@H]3([C]([C@@H](C(C)=O)CC3)(C)C1))(C[C@H](C)C=1[C]2(CCC(C1)=O)C)))=O PREGN-4-ENE TRIONE

N([P@@]([N](CCCl)CCCl)(OCCCl)=O)CCCO DEFOSFAMIDE

C1[N]([P](OCC1)(NCCCl)=O)CCCl IFOSFAMIDE

c1(c2c(c(c(OC)cc2)OC)O)c(c(c2nc3c(C(C(OC)=C(C3=O)N)=O)cc2)nc(c1C)C(O)=O)N STREPTONIGRIN

c1(nncs1)N AMINOTHIADIAZOLE

O([S](C)(=O)=O)CCNC[CH]([CH](CNCCO[S](C)(=O)=O)O)O RITROSULFAN

C(NC(CCBr)=O)NC(C=C)=O BROMACRYLIDE

c1(c(c(nc(n1)[N]1CC1)C)[N+](=O)[O-])[N]1CC1 ELDERFIELD'S PYRIMIDINE MUSTARD

c1(c(nc[nH]1)C(N)=O)/N=N\[N](C)C DACARBAZINE

C1[C@@H]2([C@@H]([C]3([C@@H](OC(=O)CC3)(C1))C)(CC[C]1([C@H]2(CC[C@]1(C)O))C)) NSC 63294

O1[CH](CCO[S]1=O)C BUTANEDIOL CYCLIC SULFITE

c12c(c3c(cccc3)nc1cccc2[N+](=O)[O-])NCCC[N](C)C NITRACRINE

c1(c(nc[nH]1)C(N)=O)/N=N\[N](CCCl)CCCl TIC-MUSTARD

C1[C]2([C@H]([C@H]3([C@H](c4c(cc(OC)cc4)CC3)(C1)))(CC[C@]2(\C(=C(\F)F)F)O))C NSC 73865

c1(C=2NCCN2)ccc(NC(c2cc(C(Nc3ccc(C=4NCCN4)cc3)=O)ccc2)=O)cc1 ISOTIC

C1=2[C@H]([C@H]3([C@]([C@@](C)(O)CC3)(C)CC2))(CCC=2[C]1(C[C@@H](C)C(C2)=O)C) HYDROXYDIMETHANDROSTADIENONE

c12c3[C@H](Cc4c1cc(OC)c(c4)Oc1c(C[C@H]4(c5c(cc(OC)c(c5)OC)CC[N]4C))cc(OC)c(c1)OC)([N](CCc3cc(c2OC)OC)C) THALICARPINE

c12c([nH]nc2)ncnc1NCCC NSC 4928

c12c(c(ncn2)S)nc[n]1CC 9-ETHYL-6-MERCAPTOPURINE

c1([N](\N=N\[N](c2ccccc2)C)C)ccccc1 SIMTRAZENE

c1(c2c(nc(n1)N)[nH]cn2)Sc1c(nc[n]1C)[N+](=O)[O-] THIAMIPRINE

C1[C@@H]2([C@@H]([C]3([C@@H](CCCC3)(C1))C)([C@H](C[C]1([C@H]2(CC[C@@H]1N))C)O)) NSC 60795

C1[CH]([CH]2CC[N](C[CH]3CO3)CC2)CC[N](C1)C[CH]1CO1 EPIPROPIDINE

c1(C(/C(=C/C(O)=O)Br)=O)ccc(OC)cc1 BROMEBRIC ACID

C([CH](C(O)=O)(N))[N](N=O)O ALANOSINE

C1[C@H]2([C@H]3([C@@H]([CH]4C(=CC(=O)CC4)C[C@H]3(C))(CC[C]2([C@H](OC(C)=O)(C1))C))) TRESTOLONE ACETATE

C1[C@H]2([C@H]3([C]([C@@H](OC(CC)=O)CC3)(CC[C@@H]2([C]2([C@@H](CCCC2)(C1))C))C)) ANDROSTANOL PROPIONATE

C1[C@H]2([C@H]([C@H]3([C]([C@@H](OC(CC)=O)CC3)(C)C1))([C@@H](CC=1[C]2(CCC(C1)=O)C)C)) 7-METHYLTESTOSTERONE PROPIONATE

c12[n](ncc1)CCN2 IMIDAZOPYRAZOLE

C1[C@@H]2([C]3(C(C=C[C@H]2([C@H]2([C](OC(=O)CC2)(C)C1)))=CC(=O)CC3)C) NSC 50903

C1([C]2([C@H]([C@H]3([C](OC(=O)CC3)(C)C1))(CCC=1[C]2(C=CC(C1)=O)C))F)=O 9-FLUOROXOTESTENOLOLACTONE

c12c3c(c4C[C@H]5(CCC[N]5Cc4c1ccc(c2OC)OC))cc(OC)c(c3)OC TYLOCREBIN-(+)

C1[C@@]23[C]([C@@H]4([C@@H]([C@H]5([C@](OC(=O)CC5)(C)CC4))(C1)))([C@H](CC(C3)=O)(SS2))C NSC 56955

c12c(C([C@@H](O[C@@H]3C[C@H](O[C@H]4C[C@@H](O[C@@H]5C[C@]([C@H](OC([CH](C)C)=O)[C@H](O5)C)(C)O)[C@H](O)[C@H](O4)C)[C@@H](O)[C@@H](O3)C)[C@@H](C1)([C@@H](C([C@H]([C@@H](C)(O))O)=O)OC))=O)c(c1c(cc(cc1c2)O[C@@H]1C[C@H]([C@@H](O[C@@H]2C[C@H]([C@@H](OC)[C@H](O2)C)O)[C@H](O1)C)OC(C)=O)O)O OLIVOMYCIN A

c12c(c(cc3c1C=C[C](O3)(C)C)OC)c(c1ccccc1[n]2C)=O ACRONINE

c1([CH](C(OCC[N]2CC[N](CCOC([CH](c3ccccc3)CC)=O)CC2)=O)CC)ccccc1 FEBUVERINE

Cl[C@]12[C@H]([C@H]3([C@]([C@@](C)(O)CC3)(C)C[C@@H]2O))(CCC=2[C]1(CCC(C2)=O)C) CHLORODIHYDROXYANDROSTENONE

C1[C@@H]2([C@@H]([C]3(C(=C(C(=O)CC3)C)C1)C)([C@H](C[C]1([C@H]2(CC[C@]1(C)O))C)O))
DIMETHYLHYDROXYTESTOSTERONE

c12c3c([C@@H](NC(C)=O)CCc1cc(O)c(c2OC)OC)cc(=O)c(cc3)OC DESMETHYLCOLCHICINE

C1([C@@H]2([C]3([C@@H](CC[C@H]2([C@H]2([C](C(=O)CC2)(C)C1)))(CC(=O)C=C3))C))=O
ANDROSTENETRIONE

O([S](C)(=O)=O)[CH]([CH]([CH]([CH](O[S](C)(=O)=O)CO[S](C)(=O)=O)O)O)CO[S](C)(=O)=O
MANNOSULFAN

C1[C@H]2([C@H]([C@H]3([C]([C@@H](OC(CC)=O)CC3)(C)C1))(CCC=1[C]2(CC(=O)C1)C)) A-NORTESTOSTERONE PROPIONATE

C1[C@H]2([C@H]([C@H]3([C]([C@@H](O)CC3)(C)C1))([C@@H](CC=1[C]2(CCC(C1)=O)C)C)) 7-METHYLTESTOSTERONE

C([C@H](NC(CC[C@@H](C(O)=O)N)=O)CCC(C=[N+]=[N-])=O)(N[C@@H](CCC(C=[N+]=[N-])=O)C(O)=O)=O
AZOTOMYCIN

c12c(C[N](CCCl)CC)csc1ccc(c2)Br MITOTENAMINE

c12[n@@]([C@@H]3O[C@H](CO)[C@H]([C@H]3O)O)cnc1c(ncn2)NC\C=C(/C)C RIBOPRINE

c1(c2cc(c(Cl)cc2)Cl)c(nc(N)nc1N)C METOPRINE

c1([C](c2ccccc2)(OC(N[CH]2CCCCC2)=O)C#C)ccccc1 ENPROMATE

C1[C@H]2([C@H]3([C@@H]([C]4(C(=CC(=O)CC4)CC3)C)(CC[C]2([C@H](OC(CC)=O)[C@H]1F)C))) 16-FLUOROTESTOSTERONE PROPIONATE

C1([C@H]2([C@@H]3([C@H]([C@H]2(C(=O)N1))([C@H]1([C@@H]2(C(=O)NC([C@H]2([C@H]3(C=C1)))=O))))))=O
MITINDOMIDE

N([CH]1CCCCC1)C([N](CCCl)N=O)=O LOMUSTINE

O([S](C)(=O)=O)CCCNCCCO[S](C)(=O)=O IMPROSULFAN

c1([n](C[CH](COC)O)ccn1)[N+](=O)[O-] MISONIDAZOLE

c1([n](C[CH](CO)O)ccn1)[N+](=O)[O-] DESMETHYLMISONIDAZOLE

N([CH]1C(NC(=O)CC1)=O)C([N](CCCl)N=O)=O PCNU

N([CH]1CC[CH](C)CC1)C([N](CCCl)N=O)=O SEMUSTINE

C1[C@@H]2([C@@H]([C]3(C(=CC(=O)CC3)C1)C)(CC[C]1([C@H]2(CCC[C@@H]1OC(CC)=O))C)) D-HOMOSTESTOSTERONE PROPIONATE

c12[C@]34[C@@H]5([C]([C@@H](OC(C)=O)[C]([C@@H]3[N@](C)c1cc(OC)c(c2)[C]1(c2c(c3ccccc3[nH]2)CC[N]2C[C@H](C1)([CH]([C@H](CC)C2)O))C(OC)=O)(C(OC)=O)O)(C=CC[N]5CC4)CC) VINROSIDINE

C1[C@H]2([C@H]3([C@@H](c4c(cc(O)cc4)CC3)(CC[C]2(C(=O)O1)C))) NSC 93236

[Pt](Cl)Cl.N.N CISPLATIN

c12c=3c(c(OC)c(c1ccc(n2)c1c(c(c2c(c(c(OC)cc2)OC)O)c(C)c(n1)C(O)=O)N)O)=N[C](N3)(C)C AZASTREPTONIGRIN

C1[C@H]2([C@H]([C@H]3([C]([C@@H](OC(CC)=O)CC3)(C)C1))(CC[C@@H]1([C]2(C=CC(C1)=O)C)))
ANDROSTENONOL PROPIONATE

c12c([n](C)c(n2)CCCC(O)=O)ccc(c1)[N](CCCl)CCCl BENDAMUSTINE

c12c(c([n](C)c(n1)N)=S)nc[nH]2 METHIOGUANINE

c1(cc(c(O)cc1)O)C(C[N](C)C)=O N-METHYLADRENALONE

C1[C@H]2([C@H]([C@H]3([C]([C@@](C)(O)CC3)(C)C1))([C@H](CC=1[C]2(CCC(C1)=O)C)(C)))
CALUSTERONE

c1(C(NNC(C)=O)=O)ccccn1 AZAPICYL

c12c([N](CCCl)CCCl)cc(C)c(c1cccc2)OC MITOCLOMINE

c1([n]([CH]2CCCO2)cc(F)c([nH]1)=O)=O TEGAFUR

c12[n@@]([C@@H]3O[C@H](CO)[C@H]([C@H]3O)O)cc(C(N)=O)c1c(ncn2)N SANGIVAMYCIN

c1(c2cc(c(Cl)cc2)Cl)c(nc(N)nc1N)CC ETOPRINE

c1(ccc(O)cn1)/C=N/NC(N)=S NSC 107392

C1[C@@H]2([C@@H]([C]3([C@@H](C[C@H]4[C@@H](C3)S4)(C1))C)(CC[C]1([C@H]2(CC[C@@H]1O[C]1(CCCC1)OC))C)) MEPITIOSTANE

C([N]1CC1)[CH](OC(C)=O)C=C ALPHA-VINYLAZIRIDINOETHYL ACETATE

C([C@H]([C@H](C[N]1CC1)O)O)[N]1CC1 BIS(AZIRIDINYL)BUTANEDIOL

c12c(/C=C\c3ccc([N](C)C)cc3)ccnc1cccc2DIMETHAMINOSTYRYLQUINOLINE

O1[P]([N](CCCl)CCCl)([N](CCC1)CCCl)=O TROFOSFAMIDE

C1[C@@H]2([C@@H]([C]3(C(=CC(=O)C=C3)C1)C)([C@H](C[C]1([C@H]2(CC[C@]1(C)O))C)(O))) NSC 59614

C1[C]2([C@H]([C@H]3([C@H]([C]4(C(=CC(=O)CC4)CC3)C)(C1)))(C[C@H]([C@@H]2OC(CC)=O)OC(CC)=O))C
HYDROXYTESTOSTERONE PROPIONATE

C1[C@H]2([C@H]([C@H]3([C]([C@@H](OC(CC)=O)CC3)(C)C1))(CCC=1[C]2(C[C@@H](OC(CC)=O)C(C1)=O)C)) NSC 12204

c1([n](CC(NCCO)=O)ccn1)[N+](=O)[O-] ETANIDAZOLE

c12c([C@H](C[C](C2)(C(CO)=O)O)O[C@H]2C[C@@H]([C@H](O)[C@@H](O2)C)N)c(c2C(c3c(cccc3C(c2c1O)=O)OC)=O)O DOXORUBICIN

O[CH]([CH]([CH]1CO1)O)[CH]1CO1 DIANHYDROGALACTITOL

C1[CH]([CH]([CH](C)C[C]1(OC(C)=O)C)O)[CH](C[CH]1CC([N](C)C(C1)=O)=O)O GLUTARIMIDE-NSC

C1[N+]2(CC[N+]3(C1)CC[N](C[CH](CCl)O)CC3)CC[N](C[CH](CCl)O)CC2.[Cl-].[Cl-] PROSPIDIUM CHLORIDE

c1(c2cc3c([nH]c(n3)c3ccc(O)cc3)cc2)nc2cc([N]3CC[N](C)CC3)ccc2[nH]1 PIBENZIMOL

c12[n]([C@H](O[C@H](CO)(C=O))(C=O))cnc1c(nc[nH]2)=O INOSINE DIALDEHYDE

N([C@@]1([C@@]([C@@](COC(c2c(cccc2O)C)=O)(O)[C@H]([C@@H]1N)Nc1cc(ccc1)C(C)=O)(C)O)[C@@H](C)(O))C([N](C)C)=O PACTAMYCIN

C1(=C(C(C([N]2CC2)=C(C1=O)C)=O)[CH](COC(N)=O)OC)[N]1CC1 CARBOQUONE

c12c(c(c(C\C=C(\CCC(O)=O)C)c(c1C)OC)O)C(=O)OC2 MYCOPHENOLIC ACID

C1=2[CH]([CH]3[CH](C(=O)OC3=O)CC2OC(C)=O)C[C@H]2([C@H]3([C@@H]([C]4(C(C[C@@H](OC(C)=O)CC4)=CC3)C)(CC[C]12C))) NSC 61716

C([N]1CC(NC(C1)=O)=O)[CH]([N]1CC(NC(C1)=O)=O)C RAZOXANE

N([CH]1[C@H]([C@@H]([C@@H](O)CO1)O)O)c1ccc(C(O)=O)cc1 BENAXIBINE

C1[C@H]2([C@H]3([C]([C@](C(COC(CCCc4ccc([N](CCCl)CCCl)cc4)=O)=O)(O)CC3)(C[C@@H]([C@@H]2([C]2(C(=CC(=O)C=C2)C1)C))O)C)) PREDNIMUSTINE

c12[C@H]([C@@H]3([C@H](COC3=O)([C@@H](c1cc1OCOc1c2)O[C@@H]1[C@H]2[C@@H](O[CH](c3cccs3)OC2)[C@H](O)[C@H](O1)O)))c1cc(c(O)c(c1)OC)OC TENIPOSIDE

O1[C@@H](O[C@H](c2ccccc2)(C#N))[C@@H]([C@@H](O)[C@@H]([C@H]1CO[C@@H]1(O[C@@H]([C@@H](O)[C@@H]([C@H]1O)O)CO))O)O AMYGDALIN(D)

O1[C@H]([C@@H]2([C@@H](C)(O2)))([C@@H](C=CC1=O)OC(C)=O) ASPERLIN

C1[C@H]2([C@H]([C@H]3([C]([C@@H](O)CC3)(C)C1))([C@@H](CC=1[C]2(C=CC(C1)=O)C)C)) DEHYDRO-7-METHYLTESTOSTERONE

c12c([nH]c(n2)NC(OC)=O)ccc(c1)C(c1cccs1)=O NOCODAZOLE

O1[C@@H]2([C@@H](Oc3[n]2ccc(n3)=N)([C@@H](O)([C@H]1(CO)))) ANCITABINE

c1(nc(nc(n1)NC)[N](C)C)[N](C)C PENTAMETHYLMELAMINE

O1[C]2([C@H]1([C@@H]([C@@H]1(C[C]([C@@H](C=CC=C(Cc3cc([N](C(C[C@H]2(OC([C@@H]([N@](C(C)=O)C)(C))=O))=O)C)c(c(OC)c3)Cl)C)OC)(NC(O1)=O)O))C))C MAYTANSINE

c12c(C(C(O)=C(C1=O)C\C=C(/Cl)Cl)=O)cccc2 DICHLORALLYL LAWSONE

c1([n@@]([C@H]2(CCCO2))cc(F)c([nH]1)=O)=O FTORAFUR

N=1[P](=N[P]([N]2CC2)([N]2CCOCC2)=N[P]1([N]1CC1)[N]1CC1)([N]1CC1)[N]1CC1 FOTRETAMINE

O1[C@@H]2[n@@]3c(nc(=N)c(c3)F)O[C@@H]2[C@H](O)[C@@H]1CO FLUROCITABINE

N([P]1([N](CCCO1)CCCl)=O)CCO[S](C)(=O)=O SUFOSFAMIDE

C1[CH]([CH](c2ccc(F)cc2)O)CC[N](C1)CCCC(c1ccc(F)cc1)=O DIHYDROLENPERONE

c12c3c([CH](NC(C)=O)CCc2cc(c(c1OC)OC)OC)cc(O)cc3 ACETYLCOLCHINOL

O1[C@@H]([N@@]2C(CC(=O)C=C2)=O)[C@@H]([C@@H](O)[C@H]1CO)O DEAZAURIDINE

C1([C@H]([C@H]2(CC[C@H](Cl)CN2))(NC(=O)[C@@H](N1)([C@H]1(CC[C@H](Cl)CN1))))=O PIPERAZINEDIONE

c1([N]2[C](N=C(N)N=C2N)(C)C)cc(c(OCc2cc(ccc2)C([N](C)C)=O)cc1)Cl.C([S](O)(=O)=O)C TRIAZINATE

C1[C@]23[C@@H]4([C@]([C@@H](O)[C@@H]([C@@H]2([C]2([C@H](C(=C(O)C(C2)=O)C)(C[C@H]3(OC([C@@H]4OC(/C=C(\[CH](C)C)C)=O)=O)))C))O)(C(OC)=O)O1) BRUCEANTIN

C1([C]2(C(O[Pt]O1)=O)CCC2)=O.N.N CARBOPLATIN

C1[N+]2([C@H](C(COC([C]([CH](C)C)([C@H](C)O)O)=O)=C1)([C@H](O)CC2))[O-] INDICINE-N-OXIDE

c12c(c([nH]c(c1)N)=O)nc[nH]2 DEZAGUANINE

C1[C]2(CC[Ge](CC)(CC)CC2)CC[N]1CCC[N](C)C SPIROGERMANIUM

N([P]([N]1CC1)([N]1CC1)=O)c1c2c(nc(n1)[N](C)C)nc[n]2C PUMITEPA

c1(c(nc(C)nc1)N)CNC([N](CCCl)N=O)=O NIMUSTINE

C1(=C(C(C([N]2CC2)=C(C1=O)NC([CH]1CCCCC1)=O)=O)NC([CH]1CCCCC1)=O)[N]1CC1 NSC 51915

c12c3c(cc(=O)c(cc3)OC)[C@@H](N)CCc2cc(c(c1OC)OC)OC.C([C@@H]([C@H](C(O)=O)(O))(O))(O)=O DESACETYLCOLCHICINE TARTRATE

c12c(c(C(O)=O)n[n]1Cc1c(cc(Cl)cc1)Cl)cccc2 LONIDAMINE

c12c(c(C(O)=O)n[n]1Cc1c(cc(Cl)cc1)C)cccc2 TOLNIDAMINE

c1(c2c(nc3c1cccc3)cccc2)Nc1c(cc(N[S](C)(=O)=O)cc1)OC AMSACRINE

N([CH](CC(O)=O)C(O)=O)C(C[P](O)(O)=O)=O SPARFOSIC ACID

c12c(ccc(c1C)CNc1cc(c(OC)c(c1)OC)OC)nc(N)nc2N TRIMETREXATE

c12c([N]([CH](CCNc3ccc(C(N[C@@H](CCC(O)=O)C(O)=O)=O)cc3)CN1)C)c(nc([nH]2)N)=OKETOTREXATE

c12[C@]34[C@@H]5([C]([C@@H](O)[C@@]([C@@H]3([N](C)c1cc(OC)c(c2)[C@@]1(c2c(c3ccccc3[nH]2)CC[N]2C[C@@H](C1)(C[C@](CC)(O)C2))C(OC)=O))(C(N)=O)O)(C=CC[N]5CC4)CC) VINDESINE

c12[C@]34[C@@H]5([C]([C@@H](OC(C)=O)[C@@]([C@@H]3([N](C=O)c1cc(OC)c(c2)[C@@]1(c2c(c3ccccc3[nH]2)CC[N]2C[C@@H](C1)[C@@H]1([C](C2)(CC)O1))C(OC)=O))(C(OC)=O)O)(C=CC[N]5CC4)CC) VINFORMIDE

c12c([C@H](C[C@@](C2)(\C(=N/NC(c2ccccc2)=O)C)O)(O[C@H]2C[C@@H]([C@H](O)[C@@H](O2)C)N))c(c2C(c3c(cccc3C(c2c1O)=O)OC)=O)O ZORUBICIN

N([C@@H]1[C@H]([C@@H]([C@@H](CO)O[C@@H]1O)O)O)C([N](CCCl)N=O)=O CHLOROZOTOCIN

c12c3c([n](CC[N](C)C)c(c1cccc2cc(c3)[N+](=O)[O-])=O)=O MITONAFIDE

c12c3c([n](CC[N]4CCCC4)c(c1cccc2cc(c3)[N+](=O)[O-])=O)=O PINAFIDE

O1[CH]2[CH]([CH](COC(c3ccc([N+](=O)[O-])cc3)=O)O[CH]2NC([N](CCCl)N=O)=O)O[C]1(C)C BOFUMUSTINE

C1[C@H]2([C@H]([C@H]3([C](OC(=O)CC3)(C)C1))([C@@H](CC=1[C]2(CCC(C1)=O)C)O)) 7-HYDROXYTESTOLOLACTONE

O1[C@@H]([n@]2c(nc(NC(CCCCCCCCCCCCCCCCCCCCC)=O)cc2)=O)[C@H]([C@@H](O)[C@H]1CO)O ENOCITABINE

c12c([C@H](C[C](C2)(C(CO)=O)O)O[C@@H]2C[C@@H]([C@@H](O)[C@@H](O2)C)N)c(c2C(c3c(cccc3C(c2c1O)=O)OC)=O)O EPIRUBICIN

C1([C]2(NC(=O)[N]1CC[N](CCCl)CCCl)CCCCC2)=O SPIROMUSTINE

c12c([C@H](C[C]([C@@H]1C(OC)=O)(CC)O)O[C@H]1C[C@@H]([C@H](O[C@H]3C[C@@H]([C@H](O[C@@H]4O[C@H](C(=O)CC4)C)[C@@H](O3)C)O)[C@@H](O1)C)[N@](C)C)c(c1C(c3c(cccc3C(c1c2)=O)O)=O)O ACLARUBICIN

C1(=C(C(C([N]2CC2)=C(C1=O)NC(OCC)=O)=O)NC(OCC)=O)[N]1CC1 DIAZIQUONE

C1([C@H]2([C@H]([C@H]3([C]([C@@H](OC(CC)=O)CC3)(C)C1))(C\C(=C(\Br)Br)C=1[C]2(CCC(C1)=O)C)))=O NSC 56940

c12c(c(c3cc[n+](cc3c2C)C)C)[nH]c2c1cc(O)cc2.C(C)(=O)[O-] ELLIPTINIUM ACETATE

c1(C(Nc2c(cccc2)N)=O)ccc(N)cc1 DINALINE

c12c(c(c3C(c4ccccc4C(c3c2O)=O)=O)O)C[C@](C(C)=O)(O)C[C@@H]1O[C@H]1C[C@@H]([C@H](O)[C@@H](O1)C)N IDARUBICIN

O1[C@@H]([C@H]([C@H](O)[C@H]([C@H]1OC)O)O)CNC([N](CCCl)N=O)=O RANIMUSTINE

C1([CH]2[N](C(=O)N1)C2)=N IMEXON

c1([n](c([n](C[C@@H]2(CO2))c([n]1C[C@H]1(CO1))=O)=O)C[C@H]1(CO1))=O TEROXIRONE

c1([C@@H]2O[C@H](CO)[C@H]([C@H]2O)O)nc(C(N)=O)cs1 TIAZOFURIN

C([N](CCCl)N=O)(NCCO)=O ELMUSTINE

c1([n](cc(F)c([nH]1)=O)C(NCCCCCC)=O)=O CARMOFUR

C1(C(O[Pt]O1)=O)=O.C1[C@H]([C@@H](CCC1)N)N OXALIPLATIN

c12c3[n@@]([C@@H]4O[C@H](CO[P](O)(O)=O)[C@H]([C@H]4O)O)cc1c(n[n](c2ncn3)C)N TRICIRIBINE PHOSPHATE

[Pt](O)(O)(Cl)Cl.C[CH](C)N.C[CH](C)N IPROPLATIN

c12c([C@H](C[C@@](C2)(C(CO)=O)O)O[C@H]2C[C@@H](C[C@@H](O2)C)N)c(c2C(c3c(cccc3C(c2c1O)=O)OC)=O)O ESORUBICIN

C1[N]([C@@]([N]2[CH](C#N)C2)(C)C)[CH]1C(N)=O AZIMEXON

C([C](F)(F)F)[C@@H]([C@H](c1ccc(O)cc1)(C))(c1ccc(O)cc1)   TERFLURANOL

c12c(c(ccc1NCCNCCO)NCCNCCO)C(c1ccccc1C2=O)=O   AMETANTRONE

O([C@H]([C@H](NC(c1nc([CH](NC[C@@H](C(N)=O)N)CC(N)=O)nc(c1C)N)=O)(C(N[C@@H]([C@H](CC(N[C@H](C(N[CH]([CH](c1nc(c2sc(C(NCCC[CH](CC(NCCCNCCCCN)=O)N)=O)cn2)cs1)O)O[C@H]1[C@@H]([C@@H]([C@H](N)[C@@H](O1)C)O)O)=O)([C@@H](C)O))=O)O)C)=O))(c1c[nH]cn1))[C@H]1[C@@H](O[C@@H]2[C@H]([C@H]([C@H](O)[C@H](O2)CO)OC(N)=O)O)[C@H]([C@H](O)[C@@H](O1)CO)O   TALISOMYCIN

c12c(c(c3ccnc(c3c2)NCCC[N](CC)CC)C)[nH]c2c1cncc2   PAZELLIPTINE

c12c(c(ccc1NCCNCCO)NCCNCCO)C(c1c(ccc(c1C2=O)O)O)=O   MITOXANTRONE

C=1([C](CCCC1C)(C)C)/C=C\C(=C/C=C\C(=C/C(Nc1ccc(O)cc1)=O)C)C   FENRETINIDE

O1[C@@H]([n@@]2c(nc(N)nc2)=O)[C@H]([C@@H](O)[C@H]1CO)O   FAZARIBINE

c12c([C@H](C[C@@](C2)(C(COC([CH](OCC)OCC)=O)=O)O)O[C@H]2C[C@@H]([C@H](O)[C@@H](O2)C)N)c(c2C(c3c(cccc3C(c2c1O)=O)OC)=O)O   DETORUBICIN

C([C](C(O)=O)([CH](F)F)N)CCN   EFLORNITHINE

c12[C@@]34[C@H]([C@]5([C@H](OC(C)=O)([C]6([C@@H]3([N](CC=C6)CC4))CC))C([N](CCCl)C(O5)=O)=O)([N](C)c1cc(OC)c(c2)[C@@]1(c2c(c3ccccc3[nH]2)CC[N]2C[C@@H](C1)C[C@](CC)(O)C2)C(OC)=O)   VINZOLIDINE

c12[C@]34[C@@H]5([C]([C@@H](OC(C)=O)([C@@]([C@@H]3([N](C=O)c1cc(OC)c(c2)[C@@]1(c2c(c3ccccc3[nH]2)CC[N]2C[C@@H](C1)C[C@@H](CC)(C2))C(OC)=O))(C(OC)=O)O))(C=CC[N]5CC4)CC)   VINEPIDINE

O([C@@H]([C@H](NC(c1nc([C@@H](NC[C@@H](C(N)=O)N)CC(N)=O)nc(c1C)N)=O)C(N[C@@H]([C@H]([C@@H](C(N[C@H](C(NCCc1nc(c2nc(C(NCCCN[C@@H](c3ccccc3)C)=O)cs2)cs1)=O)[C@@H](C)O)=O)C)O)C)=O)c1c[nH]cn1)[C@H]1[C@@H](O[C@@H]2[C@@H]([C@H]([C@H](O)[C@H](O2)CO)OC(N)=O)O)[C@H]([C@H](O)[C@@H](O1)CO)O   PEPLOMYCIN

c12c3c([n](CC[N](C)C)c(c1cccc2cc(c3)N)=O)=O   AMONAFIDE

C1[CH]([CH]2[CH](C(=O)OC2=O)C*)O[CH](*)[CH]2[CH]1C(OC2=O)=O   PYRAN COPOLYMER

C([CH](COCCCCCCCCCCCCCCCCCC)OC)O[P@@](OCC[N+](C)(C)C)(=O)[O-] EDELFOSINE

c12[C@]34[C@@H]5([C]([C@@H](OC(C)=O)[C]([C@@H]3([N](C)c1cc(OC)c(c2)[C]1(c2c(c3ccccc3[nH]2)C[N]2CC(=C[C@H](C1)C2)CC)C(OC)=O))(C(OC)=O)O)(C=CC[N]5CC4)CC) VINORELBINE

c12c3c([C@]4(O[C@@H](O3)([C@@H](O)[C@@H]([C@H]4O)[N@](C)C))C)cc(c1C(c1c(c3[C@@H](C[C@](Cc3cc1C2=O)(C)O)OC)O)=O)O   MENOGARIL

c12c(c(c(Cc3c(ccc(c3)OC)OC)cn1)C)c(nc(n2)N)N   PIRITREXIM

c1(cc(c(CO)cc1)O)[CH](C(O)=O)(N)   FORFENIMEX

C([N](CCCl)N=O)(NC[C](C)(C)C)=O   PENTAMUSTINE

c12c3c(ccc(c3)OC)[nH]c2ccc2c1c[n+](CC[N]1CC[CH]([CH]3CC[N](CC[n+]4cc5c6c7c(ccc(c7)OC)[nH]c6ccc5cc4)CC3)CC1)cc2.[Cl-].[Cl-]   DITERCALINIUM CHLORIDE

C1CCCC[C]1(CN)CN.[O-][S]([O-])(=O)=O.[Pt+2]   SPIROPLATIN

c12c([C](CC[C]2(C)C)(C)C)ccc(c1)/C(=C\c1ccccc1)C   TEMAROTENE

c12[n@@]([C@@H]3O[C@H](CO[P](O)(O)=O)[C@H]([C@@H]3O)O)cnc1c(nc(n2)F)N   FLUDARABINE PHOSPHATE

C1([N]2[CH](C([N]([CH](Cc3ccc(OC)cc3)C(O[CH]([CH](NC([CH]([N](C([CH]3[N](CCC3)C([CH](C)O)=O)=O)C)C[CH](C)C)=O)C(N[CH](C[CH](C)C)[CH](CC(O[CH](C([CH](C(N[CH]1C[CH](C)C)=O)C)=O)[CH](C)C)=O)O)=O)C)=O)C)=O)CCC2)=O   DIDEMNIN B

c1([n]([n](c(=O)[n]1C[CH]1CO1)C[CH]1CO1)C[CH]1CO1)=O   ANAXIRONE

c12c3c(ccc2nc(cc1Nc1ccc([N](C(C)=O)C)cc1)C)nc[nH]3   ACODAZOLE

O([N](C(NC)=O)C(C)=O)C(NC)=O CARACEMIDE

c12[C@]34[C@@H]5([C]([C@@H](O)[C@@]([C@@H]3([N](C)c1cc(OC)c(c2)[C@@]1(c2c(c3ccccc3[nH]2)CC[N]2C[C@H](C1)(C[C@](CC)(O)C2))C(OC)=O))(C(N[C@@H](Cc1c2c(cccc2)[nH]c1)(C(OCC)=O))=O)O)(C=CC[N]5CC4)CC) VINTRIPTOL

C(=C(/c1ccccc1)CC)(/c1ccc(OCC[N](C)C)cc1)c1cc(ccc1)O DROLOXIFENE

C([N](C(NCCCl)=O)N=O)CSSCCNC([N](CCCl)N=O)=O DITIOMUSTINE

c12c([n](c(=O)c(c1O)C([N](c1ccccc1)C)=O)C)cccc2 ROQUINIMEX

c1(c(c2ccc(O)cc2)sc2c1ccc(c2)O)C(c1ccc(OCC[N]2CCCCC2)cc1)=O RALOXIFENE

c1([C@@]2(O[C@@H](CSc3ccc(NC(OCC)=O)cc3)(CO2))C[n]2ccnc2)c(cc(Cl)cc1)Cl TUBULOZOLE

c12[n](c([n](C)nn2)=O)cnc1C(N)=O TEMOZOLOMIDE

c12[n](c([n](CCCl)nn2)=O)cnc1C(N)=O MITOZOLOMIDE

[Ti](O\C(c1ccccc1)=C/C(C)=O)(O\C(c1ccccc1)=C/C(C)=O)(OCC)OCC BUDOTITANE

C([N](CCCl)N=O)(NCC[S@@]([N](C)C)(=O)=O)=O TAUROMUSTINE

C1[N+]2(CC[N+]3(C1)CC[N](C(CCBr)=O)CC3)CC[N](C(CCBr)=O)CC2.[Cl-].[Cl-] DIBROSPIDIUM CHLORIDE

c12c(c(cc(o2)c2ccccc2)=O)cccc1CC(O)=O FLAVONEACETIC ACID

C([CH]([C](\C=C\[CH]1OC(C=CC1)=O)(C)O)O[P](O)(O)=O)[CH](\C=C\C=C/C=C\CO)O FOSTRIECIN

N1[P@]([N@](CCCl)CCCl)(OCC[C@H]1SCC[S](O)(=O)=O)=O MAFOSFAMIDE

C(=C(/c1ccccc1)CCCl)(/c1ccc(OCC[N](C)C)cc1)c1ccccc1 TOREMIFENE

C\1[C@H]2[C@@H](CC1=C\COCCCC(O)=O)(C[C@H]([C@@H]2C#C[C@H]([C@H](CC#CCC)(C))(O))(O)) EPTALOPROST

O([P]([CH](NC([N](CCCl)N=O)=O)C)(OCC)=O)CC FOTEMUSTINE

c1(c2ccc(c3c(cccc3)F)cc2)nc2c(cc(F)cc2)c(c1C)C(O)=O BREQUINAR

c12c(c3c(cccc3)c(c2)CN[C@@](CO)(CO)C)ccc2c1cccc2 CRISNATOL

c12c([C@H]([C@](CC)(O)C[C@@H]1(O[C@H]1C[C@@H]([C@H](O[C@H]3C[C@H]4[C@H](O[C@H]5O[C@@H](C)C(C[C@@H]5O4)=O)[C@@H](O3)C)[C@@H](O1)C)[N@](C)C))(O[C@H]1C[C@@H]([C@H](O)[C@@H](O1)C)[N@@](C)C))c(c1C(c3cccc(c3C(c1c2O)=O)O)=O)O RODORUBICIN

C([CH]1C(NC(=S)NC1=O)=O)(Nc1ccccc1)=O MERBARONE

N([C@H](C(NCCCCNCCCN)=O)O)C(CCCCCCNC(N)=N)=O DEOXYSPERGUALIN

c12c3c4c(c(ccc4O)O)c(c1c(ccc2[n](CCNCCO)n3)NCCCN)=O PIROXANTRONE

S(CCO)CCO THIODIGLYCOL

O1[C@@H]([n@]2c(nc(N)nc2)=O)C[C@@H](O)[C@H]1CO DECITABINE

C([C@H]([C@H](Cc1cc(c(O)cc1)O)(C))(C))c1cc(c(O)cc1)O MASOPROCOL

O([P](OCC[N+](C)(C)C)(=O)[O-])CCCCCCCCCCCCCCCC MILTEFOSINE

C1=2[N](C(=O)c3c1cccc3)Cc1cc(N)ccc1N2 DANIQUIDONE

C1[C@H]2([C@H]3([C@@H](c4c(cc(OC(c5ccccc5)=O)cc4)CC3)(CC[C]2([C@H](OC(COC(CCCc2ccc([N](CCCl)CCCl)cc2)=O)=O)(C1))C))) ATRIMUSTINE

c12c(ncc(n1)C[CH](c1ccc(C(N[C@@H](CCC(O)=O)(C(O)=O))=O)cc1)CC)nc(N)nc2N EDATREXATE

c1([N](CCCl)CCCl)cc(C[C@@H](C(N[C@H](C(OCC)=O)CCSC)=O)NC([C@H](Cc2ccc(F)cc2)N)=O)ccc1 AMBAMUSTINE

C1[C@@H]2([C@H]([N](C[C@H](C2)(N[S]([N](CC)CC)(=O)=O))CCC)(Cc2cccc(c12)O)) QUINAGOLIDE

O1[C@@H]([n@@]2c(nc(N)cc2)=O)[C]([C@@H](O)[C@H]1CO)(F)F GEMCITABINE

c12c3c4c(=O)oc1c1c(cccc1O[C@H]1[C@H](O[C@@H]5[C@@H]([C@H]([C@@H](O)[C@H](O5)C)OC)N)[C@@]([C@@H](O)[C@H](O1)C)(C)O)c(c2c(=O)oc3ccc4C)O ELSAMITRUCIN

C1[C@H]2([C@H]3([C@@H]([C]4([C@H](NC(=O)C=C4)(CC3))C)(CC[C]2([C@H](C(N[C](C)(C)C)=O)C1)C))) FINASTERIDE

C1[C@H]([C@@H]([C@H](O[C@@H]1OC)CO)O)NC([N](CCCl)N=O)=O ECOMUSTINE

C1([N](C(C[N](C1)CC[N]1CC([N](COC(OC[CH](C)C)=O)C(C1)=O)=O)=O)COC(OC[CH](C)C)=O)=O SOBUZOXANE

c12[n]([CH](c3ccc(C#N)cc3)CCC1)cnc2 FADROZOLE

C([C@@H]1[C@@H]([C@@H]([C@@H](O)[CH](O1)O)O)O)[N](CCCl)CCCl GALAMUSTINE

c12c(C[C@@H](CCc3ccc(C(N[C@@H](CCC(=O)[O-])C(=O)[O-])=O)cc3)CN1)c([nH]c(n2)N)=O.[Na+].[Na+] LOMETREXOL SODIUM

c12c(ccc(c1)[S](NC(Nc1ccc(Cl)cc1)=O)(=O)=O)CCC2 SULOFENUR

C1=2[C@@]3([C@@H](C[N]1C(c1cc4cc(NC(c5cc6ccccc6o5)=O)ccc4[nH]1)=O)C3)c1c([nH]cc1C)C(C2)=O ADOZELESIN

c1([C@@H]([C@H](CN)(Cl))(O))nc(N)[nH]c1 GIRACODAZOLE

C1([C]2(C(O[Pt]O1)=O)CCC2)=O.C([C](CN)(CO)CO)N ZENIPLATIN

c12c3[n](Cc4ccccc4[CH]3C)cc1C(c1c(O[P](OCc3ccccc3)(O)=O)cccc1C2=O)=O FOSQUIDONE

C1[C]2([C@H]3([C@@H]([C]4([C](C(=C(C)[C@H](C4)OC([C@@H]([C@H](c4ccccc4)NC(O[C](C)(C)C)=O)O)=O)[C@H](C([C]3([C@@H](O)C[C@@H]2(O1))C)=O)O)(C)C)O)OC(c1ccccc1)=O))OC(C)=O DOCETAXOL

c1(cc(c(O)c(c1)O)[N+](=O)[O-])/C=C(/C(C)=O)C(C)=O NITECAPONE

c12c(c([n]3Cc4c(c3c2)nc2ccc(c(c2c4)C[N](C)C)O)=O)COC([C]1(CC)O)=O TOPOTECAN

C=1([C@@H]([C@H]2(O[C@@](c3ccccc3)OC2))(OC(=O)C1O))O ZILASCORB

c12[CH]3[CH](OC(c1cc(O)c(c2O)OC)=O)[CH]([CH](O)[CH](O3)CO)O BERGENIN

C1[C@H]2([C@H]([C@H]3([C](C(=O)CC3)(C)C1))(CCC=1[C]2(CCC(C1O)=O)C)) FORMESTANE

c12[n@@]([C@@H]3O[C@H](CO)[C@H](C3)O)cnc1c(nc(n2)Cl)N CLADRIBINE

O1[C@@H]([n@@]2c(nc(N)cc2)=O)CC[C@H]1CO DIDEOXYCYTIDINE

C([N]1CC(NC(C1)=O)=O)[C@@H]([N]1CC(NC(C1)=O)=O)C DEXRAZOXANE

N([C@H](C(NCC(O)=O)=O)(CSSC[C@H](NC(CC[C@@H](C(O)=O)(N))=O)(C(NCC(O)=O)=O)))C(CC[C@@H](C(O)=O)(N))=O OXIGLUTATIONE

c12c([n+](nc([n+]1[O-])N)[O-])cccc2 TIRAPAZAMINE

C1[C]2([C@H]([C@H]3(C(=C4C(=CC(=O)CC4)CC3)C1))(CC[C@@]2(C([C@H](C)(O))=O)C))C TRIMEGESTONE

C1=2[C]([C@@H]3([C@H]([C@H]4([C@](C(=O)CC4)(C)CC3))(CC1)))(CCC(C2)=O)CC#C PLOMESTANE

c12[C@]34[C@@H]5([C]([C@@H](O)[C]([C@@H]3([N](C)c1cc(OC)c(c2)[C]1(c2c(c3ccccc3[nH]2)CC[N]2C[C@H](C1)(C[C](CC)(O)C2))C(OC)=O))(C(N[CH](C(OCC)=O)[CH](CC)C)=O)O)(C=CC[N]5CC4)CC) VINLEUCINOL

c12c3c4c(c(ccc4)O)c(c1c(ccc2[n](CCNCCO)n3)NCCNCCO)=O LOSOXANTRONE

C1[CH]2[CH](CC1=O)CC[CH]2CCCC[CH](CC)OC CIOTERONEL

c12c3c4c(c(ccc4O)O)c(c1c(ccc2[n](CCNCCO)n3)NCCNC)=O TELOXANTRONE

C1(CO[Pt]O1)=O.N.N NEDAPLATIN

c12c3[n](c(c4COC([C@@](c4c3)(CC)O)=O)=O)Cc1c(c1cc(OC([N]3CC[CH]([N]4CCCCC4)CC3)=O)ccc1n2)CC IRINOTECAN

C1[C]2(CC1)C(O[Pt]OC2=O)=O.C1[C@@H](NCC1)(CN) MIBOPLATIN

C(CCNC(N)=N)[C@H](C(O)=O)(N).C([S](O)(=O)=O)CS ARGIMESNA

C1[C]2(CC1)C(O[Pt]OC2=O)=O.C([C@H](CN)(C))CN SEBRIPLATIN

C([n]1c([n](cc(c1=O)F)COCC)=O)(c1cc(C(Oc2c(ccc(n2)OC(c2ccccc2)=O)C#N)=O)ccc1)=O EMITEFUR

C1([C]2(C(O[Pt]O1)=O)CCC2)=O.C1[C](CCOC1)(CN)CN ENLOPLATIN

c1(cc(NC(c2cc(NC(c3ccc([N](CCCl)CCCl)cc3)=O)c[n]2C)=O)c[n]1C)C(Nc1cc(C(NCCC(N)=N)=O)[n](c1)C)=O TALLIMUSTINE

c12[C@H]([C@@H]3([C@H](COC3=O)([C@@H](c1cc1OCOc1c2)O[C@@H]1O[C@H]2[C@@H](O[C@H](C)OC2)[C@@H]([C@H]1O)O)))c1cc(c(O[P](O)(O)=O)c(c1)OC)OC ETOPOSIDE PHOSPHATE

c12c([N](C(c3[nH]c4ccc(cc4c3)NC(c3oc4cc([N](CC)CC)ccc4c3)=O)=O)C[C@H]2CCl)cc(OC(Nc2ccccc2)=O)c2c1c(c[nH]2)C CARZELESIN

c1([C]2(C(NC(=O)CC2)=O)CC)ccncc1 ROGLETIMIDE

c12[C@]34[C@@H]5([C]([C@@H](O)([C@@]([C@@H]3([N](C)c1cc(OC)c(c2)[C@@]1(c2c(c3ccccc3[nH]2)CC[N]2C[C@H](C1)(C[C@](CC)(O)C2))C(OC)=O))(C(N[C@@H]([P](OCC)(OCC)=O)([CH](C)C))=O)O))(C=CC[N]5CC4)CC) VINFOSILTINE

O1[C@@H]([n@]2c(nc(NC(c3cc(c(OC)c(c3)OC)OC)=O)c(c2)F)=O)[C@@H]([C@@H](O)[C@H]1C)O GALOCITABINE

c12c3c(c(cnc3NCCC[N](C)C)C)[nH]c2ccc2c1ccc(c2)O INTOPLICINE

c12c([N](C(c3cc4cc(NC(Nc5cc6c([nH]c(c6)C([N]6c7c(c8c([nH]cc8C)c(c7)O)[C@H](C6)CCl)=O)cc5)=O)ccc4[nH]3)=O)C[C@H]2CCl)cc(O)c2c1c(c[nH]2)C BIZELESIN

c1([CH](c2ccc(Cl)cc2)[n]2cncn2)cc2c(nn[n]2C)cc1 VOROZOLE

C1[C@H]([C@@H](C1)(CN))(CN).C1([C@@H](O[Pt]O1)(C))=O LOBAPLATIN

N1[P]([N](CCCl)CCCl)(OCCC1)=O CYCLOPHOSPHAMIDE

c1(c([nH]c(=O)[nH]c1=O)C)/C=C\C(N[C@H](C[S](CSC)=O)(CO))=O SPARSOMYCIN

c1(C(/C(=C\C(=O)[O-])Br)=O)ccc(OC)cc1.[Na+] ANISOYLBROMACRYLIC ACID, NA

C([N](C=O)O)C(=O)[O-].[Na+] HADACIDIN NA SALT

C(=C(/c1ccccc1)CC)(/c1ccc(OCC[N](C)C)cc1)c1ccccc1 TAMOXIFEN

c1(C(C[S](C)=O)=O)ccccn1 OXISURAN

C1[C@H]2([C@H]3([C@@H](c4c(cc(OC([N](CCCl)CCCl)=O)cc4)CC3)(CC[C]2([C@H](O[P](=O)([O-])[O-])(C1))C))).[Na+].[Na+] ESTRAMUSTINE PHOSPHATE NA

[Pt](Cl)(Cl)(Cl)Cl.C1[C@H]([C@@H](CCC1)N)N ORMAPLATIN

[Pt](Cl)(Cl)(Cl)Cl.C1[C@H]([C@@H](CCC1)N)N DEXORMAPLATIN

N1[P@]([N@@](CCCl)CCCl)(OCC[C@@H]1OO)=O PERFOSFAMIDE

c1(ccc(/N=N\[N](C)C)cc1)C(O)=O CB-10-277

C(CCCCCCCCCCCCCCC)[C@@H]([C@H](CO)N)O SAFINGOL

N([C@H]([C@H](CCCCCCCCCCCCCCC)O)CO)C(C)=O CEDEFINGOL

c1(c(ccc(c1)NC([C](C[S](c1ccc(F)cc1)(=O)=O)(C)O)=O)C#N)[C](F)(F)F BICALUTAMIDE

c1(C(c2ccc(Cl)cc2)=O)c(cc(C[n]2c(c(C(N)=O)nn2)N)cc1Cl)Cl NSC-609974

c12c3c(cc4cc(N)ccc4n3)C[n]1c(c1COC([C@@](c1c2)(CC)O)=O)=O 9-AMINOCPT

c1([CH](c2ccc(C#N)cc2)[n]2cncn2)ccc(C#N)cc1 LETROZOLE

c1(cc(c(O)cc1)O)/C=C\C(OCCc1ccccc1)=O CAFFEIC ACID PHENETHYL ESTER

c12c([C](CC[C]2(C)C)(C)C)ccc(c1)/C(=C\c1ccc(OCC[N]2CCOCC2)cc1)C MOFAROTENE

C1=2[C](C=3NC1=CC1=NC(=Cc4c(C)c(c(C=C5N=C(C3)C(=C5CCC(OC)=O)C)[nH]4)CCC(O)=O)C(=C1C)C=C)([C@@H
](C(C(OC)=O)=CC2)C(OC)=O)C.C1=2[C](C=3NC1=CC1=NC(=Cc4c(C)c(c(C=C5N=C(C3)C(=C5CCC(O)=O)C)[nH]4)C
CC(OC)=O)C(=C1C)C=C)([C@@H](C(C(OC)=O)=CC2)C(OC)=O)C VERTEPORFIN

c1(c[n](c(=O)[nH]c1=O)COCCO)Cc1ccccc1 BENZYLACYCLOURIDINE

c1(NC(c2ccc(NC(C)=O)cc2)=O)c(cccc1)N N-ACETYLDINALINE

C1[CH]2[N]([CH](c3c(cccc13)OC)CO)[CH]([CH]1[N]([CH]2[CH](C1)C(O)=O)C)C#N CYANO-QUINOCARMYCIN

c1[n@]([C@H]2C[C@H](N=[N+]=[N-])[C@H](O2)CO)c([nH]c(c1/C=C\Br)=O)=O 3-AZIDOBRIVUDINE

O([P]([N](CCCl)CCCl)(N\N=C1/C[C]([N+](=O)[C](C1)(C)C)(C)C)=O)c1ccccc1.[Cl-] F-538

C1[C@@H]2([C@@H]([C]3(C(=CC(=O)CC3)C1)C)([C@H](C[C]1([C@H]2(CC[C@@H]1C(CO)=O))C)O))
CORTICOSTERONE

c12c(oc3c(ccc(c3n1)C(N[C@@H]1C(N[C@@H](C([N]3[C@@H](CCC3)C([N](C)CC([N@]([C@H](C(O[C@@H]1C)=O)[C
H](C)C)C)=O)=O)=O)[CH](C)C)=O)=O)C)c(c(=O)c(c2C(N[C@@H]1C(N[C@@H](C([N]2[C@@H](CCC2)C([N](C)CC([N
@]([C@H](C(O[C@@H]1C)=O)[CH](C)C)C)=O)=O)=O)[CH](C)C)=O)=O)N)C DACTINOMYCIN

O1[C@@H]([n@@]2c([nH]c(=O)c(c2)C)=O)C[C@@H](O)[C@H]1CO THYMIDINE

O1[C@@H]([n@]2c([nH]c(=O)c(c2)F)=O)C[C@@H](O)[C@H]1CO FLOXURIDINE

C1[C@@H]2([C@@H]([C]3([C@H](C[C@H](O)CC3)(C1))C)(CC[C]1([C@H]2(CCC1=O))C)) ETIOCHOLANOLONE

c12[n@@]([C@H]3[C@@H]([C@H](NC([C@H](Cc4ccc(OC)cc4)N)=O)[C@H](O3)CO)O)cnc1c(ncn2)[N](C)C
PUROMYCIN

O1[C@@H]([n@]2c([nH]c(=O)cc2)=O)[C@@H]([C@@H](O)[C@H]1CO)O URIDINE

c12c(ncc(n1)C[N](c1ccc(C(N[C@@H](CCC(O)=O)C(O)=O)=O)cc1)C)nc(N)nc2N METHOTREXATE

c12[n@]([C@@H]3O[C@H](CO)[C@H]([C@H]3O)O)cnc1c(nc(n2)N)S THIOGUANOSINE

O1[C@@H]([n@]2c(nc(N)cc2)=O)[C@H]([C@@H](O)[C@H]1CO)O CYTARABINE

c1([N](CCCl)CCCl)ccc(C[C@@H](C(O)=O)N)cc1 MELPHALAN

C(C[C@@H](C(O)=O)N)C(C=[N+]=[N-])=O DON

c12c3c([C@@H](NC)CCc2cc(c(c1OC)OC)OC)cc(=O)c(cc3)OC DEMECOLCINE

C1([CH]2[CH](SC[CH](C([N@]([C@H](C(=O)OC[C@H](C(N[C@H](C([N]2C)=O)C)=O)NC(c2nc3c(cccc3)nc2)=O)[CH]
(C)C)C)=O)[N](C([C@@H](NC([C@@H](COC([C@@H]([N@@]1C)[CH](C)C)=O)NC(c1nc2c(cccc2)nc1)=O)=O)C)=O)C)S
C)=O ECHINOMYCIN

c12[CH]([C@@H]3([C@H](COC3=O)([C@H](c1cc1OCOc1c2)O)))c1cc(c(OC)c(c1)OC)OC PODOPHYLLOTOXIN

c12c3[C@H](Cc4cc(c(OC)cc4)Oc4ccc(C[C@H]5c6c(O2)cc(OC)cc6CC[N]5C)cc4)[N](CCc3cc(c1OC)OC)C
TETRANDRINE

C1[C]([C]2([C@@H]([C@H]3([C@@H]([C]4(C(=CC(=O)CC4)C(=C3)C)C)(CC2)))(C1))C)(OC(C)=O)C(C)=O MEGESTROL ACETATE

C1[C@H]2([C@H]([C@H]3([C]([C@@H](O)CC3)(C)C1))(CCC=1[C]2(CCC(C1C)=O)C)) 4-METHYLTESTOSTERONE

c1([N](CCCl)CCCl)cc(ccc1)C[CH](C(O)=O)N METAMELFALAN

c12c3c(C(c4c(c5[C@@H](O[C@H]6[C@@H]([C@@]([C@@H](OC)[C@@H](O6)C)(OC)C)OC)C[C]([C@@H](c5cc4C3=O)C(OC)=O)(C)O)O)=O)c(O)cc1[C]1(O[C@@H](O2)([C@@H](O)[C@@H]([C@H]1O)[N@@](C)C))C NOGALAMYCIN

C1([C@]([C]2([C@@H]([C@H]3([C@@H]([C]4(C(=CC(=O)CC4)C(=C3)C)C)(CC2)))(C1))C)(OC(C)=O)C(C)=O)=C MELENGESTROL ACETATE

c12c(C[C@@H]([C@H](C([C@H]([C@H](C)O)O)=O)OC)[C@@H](C1=O)O[CH]1C[C@@H](O[CH]3C[C@H](O[CH]4C[C@]([C@@H](OC(C)=O)[C@@H](O4)C)(C)O)[C@@H](O)[C@@H](O3)C)[C@H](O)[C@H](O1)C)cc1cc(O[CH]3C[C@@H]([C@H](OC(C)=O)[C@@H](O3)C)O[CH]3C[C@@H]([C@H](OC)[C@@H](O3)C)O)c(c(c1c2O)O)C TOYOMYCIN

c12c(c([n]3Cc4c(c3c2)nc2ccccc2c4)=O)COC([C@@]1(CC)O)=O CAMPTOTHECIN

O[C@@H]([C@H]([C@@H](CBr)O)O)[C@H](CBr)O MITOLACTOL

c12c(C[C@@H]([C@@H](C([C@H]([C@@H](C)O)O)=O)OC)([C@@H](C1=O)O[C@H]1C[C@@H](O[C@H]3C[C@@H](O[C@H]4C[C@]([C@H](O)[C@H](O4)C)(C)O)[C@H](O)[C@H](O3)C)[C@H](O)[C@H](O1)C))cc1cc(O[C@H]3C[C@@H](O[C@H]4C[C@H]([C@H](O)[C@H](O4)C)O)[C@@H](O)[C@H](O3)C)c(c(c1c2O)O)CPLICAMYCIN

N([C@@H]1[C@H]([C@@H]([C@@H](CO)O[CH]1O)O)O)C([N](N=O)C)=O STREPTOZOCIN

c12c([C@H](C[C@@](C2)(C(C)=O)O)O[C@H]2C[C@@H]([C@H](O)[C@@H](O2)C)N)c(c2C(c3c(cccc3C(c2c1O)=O)OC)=O)O DAUNORUBICIN

C=1[C@]23[C](c4c(cc5OCOc5c4)CC[N]2CCC3)([C@H](OC([C](CCC[C](C)(C)O)(CC(OC)=O)O)=O)C1OC)C HOMOHARRINGTONINE

c1([C@@H]2O[C@H](CO)[C@H]([C@H]2O)O)c(c(C(N)=O)[nH]n1)O PYRAZOFURIN

c12[C@H]([C@@H]3([C@H](COC3=O)([C@@H](c1cc1OCOc1c2)O[C@@H]1O[C@H]2([C@@H](O[C@H](C)OC2)([C@@H]([C@H]1O)O)))))c1cc(c(O)c(c1)OC)OC ETOPOSIDE

O1[C@@H](CC(Cl)=N1)([C@@H](C(O)=O)N) ACIVICIN

c12c(c(c3C(c4cccc(c4C(c3c2O)=O)OC)=O)O)C[C@](C(CO)=O)(O)C[C@@H]1O[C@H]1C[C@@H]([C@H](O[C@H]2CCCCO2)[C@@H](O1)C)N PIRARUBICIN

c1(c2c(cccc2)[nH]c1)C[C@H](NC([C@H](Cc1c[nH]cn1)NC([C@@H]1CCC(N1)=O)=O)=O)C(N[C@H](C(N[C@@H](Cc1ccc(O)cc1)C(N[C@@H](C(N[C@H](C(N[C@H](C([N]1[C@@H](CCC1)C(NCC)=O)=O)CCCNC(N)=N)=O)C[CH](C)C)=O)C[CH](C)C)=O)=O)CO)=O LEUPROLIDE

c12c(C[C@@H](NC([C@@H](Cc3ccc(O)cc3)NC([C@@H](NC([C@H](Cc3c4c(cccc4)[nH]c3)NC([C@H](Cc3c[nH]cn3)NC([C@H]3NC(=O)CC3)=O)=O)=O)CO)=O)=O)C(N[C@H](C(N[C@H](C([N@]3[C@@H](CCC3)C(NCC(N)=O)=O)=O)CCCNC(N)=N)=O)C[CH](C)C)=O)c[nH]c1cccc2 TRIPTORELIN

c1(c2c(cccc2)[nH]c1)C[C@@H]1NC([C@@H](Cc2ccc(O)cc2)NC([C@@H](NC([C@@H](Cc2ccccc2)N)=O)CSSC[C@H](NC([C@@H](NC([C@@H](NC1=O)CCCCN)=O)[C@@H](C)C)=O)C(N[C@@H](Cc1c2c(cccc2)[nH]c1)C(N)=O)=O)=O)=O VAPREOTIDE

c1(c2c(cccc2)[nH]c1)C[C@H]1NC([C@@H](Cc2ccc(O)cc2)NC([C@@H](NC([C@@H](Cc2cc3c(cccc3)cc2)N)=O)CSSC[C@H](NC([C@@H](NC([C@@H](NC1=O)CCCCN)=O)[C@H](C)C)=O)C(N[C@@H]([C@@H](C)O)C(N)=O)=O)=O)=O LANREOTIDE

c12c([C@H](C[C](C2)(C(CO)=O)O)O[C@H]2C[C@H]([N@]3C[C@H](OCC3)OC)[C@H](O)[C@@H](O2)C)c(c2C(c3c(cccc3C(c2c1O)=O)OC)=O)O NEMORUBACIN

c12c(c(c3C(c4ccccc4C(c3c2O)=O)=O)O)C[C@](C(C)=O)(N)C[C@@H]1O[C@H]1C[C@@H]([C@H](O)CO1)O AMRUBICIN

c1(cc(cc(c1)C[n]1cncn1)[C](C#N)(C)C)[C](C#N)(C)C ANASTROZOLE

C1[C@H]2([C@H]3([C@@H](c4c(cc(OC([N](CCCl)CCCl)=O)cc4)CC3)(CC[C]2([C@H](OC([C@H](C)N)=O)C1)C))) ALESTRAMUSTINE

c12c3c([n]([C@@H](CNCCNC[C@H]([n]4c(c5c6c(cc(c5)[N+](=O)[O-])cccc6c4=O)=O)C)C)c(c1cccc2cc(c3)[N+](=O)[O-])=O)=O.C[S](O)(=O)=O.C[S@@](O)(=O)=O BISNAFIDE DIMESYLATE

O1[C@@H]([n@@]2c(nc(NC(OCCCCC)=O)c(c2)F)=O)[C@@H]([C@@H](O)[C@H]1C)O CAPECITABINE

c12c(c([n]3Cc4c(c3c2)nc2cc3OCCOc3cc2c4C[N]2CC[N](C)CC2)=O)COC([C]1(CC)O)=O LURTOTECAN

c12c3c4c(cc(O)cc4)sc1c(ccc2[n](CC[N](CC)CC)n3)NCCN LEDOXANTRONE

C([C@@H]([C@@H](C(NO)=O)O)C[CH](C)C)(N[C@H](C(NC)=O)[C@](C)(C)C)=OMARIMASTAT

c12c3c(ccc1N=C(c2ccc3)N)[N](Cc1ccc([S]([N]2CCOCC2)(=O)=O)cc1)C METESIND

C1=2C(=C(C)C(N2)=Cc2[nH]c(C=C3N=C(C=c4[nH]c(=C1)c(c4C)CCC(=O)*)C(=C3C)*)c(c2C)[CH](O*)C)CCC(=O)[O-].C1=2C(=C(C)C(N2)=Cc2[nH]c(C=C3N=C(C=c4[nH]c(=C1)c(c4C)CCC(=O)[O-])C(=C3C)*)c(c2C)[CH](O*)C)CCC(=O)[O-].C=12C(=C(C)C(=N2)C=c2[nH]c(=CC3=NC(=Cc4[nH]c(C1)c(c4C)CCC(=O)[O-])C(=C3C)*)c(c2C)[CH](C)*)CCC(=O)[O-].[Na+].[Na+].[Na+].[Na+].[Na+] PORFIMER SODIUM

O([CH]1[C@@H]([CH]([C@H](O)[CH](O1)CO[C@@H]1O[CH]([C@@H](O)[C@@H]([C@H]1O)O)CO)O[CH]1O[CH]([C@@H](O*)C[C@H]1O)CO)O)[CH]1[C@@H]([CH](O[CH]([C@@H]1O)O*)CO)O SIZOFIRAN

C([CH]1[CH](*[CH](c2ccccc2)C)C(=O)NC1=O)C[CH]1[CH](C(=O)NC1=O)CC*[CH]([CH](CC[CH]([CH](CC*[CH](CC(O)=O)C(N)=O)C(O)=O)C(N)=O)C(O)=O)C(N)=O.N.N CARBETIMER

C=1([C]2([CH]([CH]3[CH]([C]4(C(C[C@@H](OC(C)=O)CC4)=CC3)C)CC2)CC1)C)c1cccnc1 ABIRATERONE

C([C@H](CCC(C=[N+]=[N-])=O)NC([C@H](C)N)=O)(N[C@H](CCC(C=[N+]=[N-])=O)C(O)=O)=O AMBOMYCIN

c12c(Cc3cccnc3)c[nH]c1c([nH]c(n2)N)=O PELDESINE

O1[C@H]([C@@H]2(C(=C(O)C(O2)=O)O))(CO[CH]1c1ccccc1) ZILASCORB (2H)

c12[n](c(cc1cccc2)C(Nc1nc(c2c(cccc2)Cl)cs1)=O)CC(O)=O LINTITRIPT

c1(c2c(nc3c1cccc3C)c(ccc2)C(N)=O)[N](c1c(cc(N[S](C)(=O)=O)cc1)OC)C ASULACRINE

C(=C(/c1ccc([CH](C)C)cc1)CC)(/c1ccc(OCC[N](C)C)cc1)c1ccc(O)cc1 MIPROXIFENE

C1CCNCC1 PIPERIDINE

C([N@]1[C@H](C([N@]2[C@@H](CCC2)C(NCc2ccccc2)=O)=O)CCC1)([C@@H]([N](C([C@@H](NC([C@@H]([CH](C)C)[N@@](C)C)=O)[C@H](C)C)=O)C)[C@H](C)C)=O CEMIDOTIN

c12c(c(c3cccc(c3[nH]2)OC)=O)cccc1C(Nc1ccc(CC[N]2Cc3c(cc(OC)c(c3)OC)CC2)cc1)=O ELACRIDAR

c12c3c([n](CCNCCCNCC[n]4c(c5c6c(ccc5)cccc6c4=O)=O)c(c1cccc2ccc3)=O)=O ELINAFIDE

c1(c([nH]c(=O)[nH]c1)=O)C#C ENILURACIL

c12c(cc(NC(OCC)=O)nc2N)N[C@@H](C)C(=N1)c1ccccc1.C([S](O)(=O)=O)CO MIVOBULIN ISETHIONATE

[O-][P](=O)([O-])C[N](CC[N](C[P](=O)([O-])[O-])C[P](=O)([O-])[O-])C[P](=O)([O-])[O-].[Na+].[Na+].[Na+].[Na+].[Na+].[153Sm+3] SAMARIUM SM 153 LEXIDROMAN PENTASODIUM

c12[C@]34[C@H]5[C]([C@@H](OC(C)=O)[C@@]([C@@H]3([N](C)c1cc(OC)c(c2)[C@@]1(c2c(c3ccccc3[nH]2)C[N]2C[CH](C[C@H](C1)C2)[C](C)(F)F)C(OC)=O))(C(OC)=O)O)(C=CC[N@@]5CC4)CC VINFLUNINE

O1[C@@H]2[C@@H]1CCC[C@@H]([C@@H]([C@H](C(=O)[C]([C@@H](CC(O[CH](C2)\C(=C\c1nc(C)sc1)C)=O)O)(C)C)C)O)C EPOTHILONE

O1[C@H]([C@@H]([C@@H]([C@H](C)C1=O)O)C)C[C@@H](\C=C/[C@@H]([C@@H]([C@H](\C=C(/C[C@H]([C@H]([C@@H]([C@H]([C@H](\C=C/C=C)C)OC(N)=O)C)O)C)C)C)O)C)O DISCODERMOLIDE

c12c3[N]([C@H](C(=O)N[C@@H](Cc1c[nH]c2ccc3)CO)[C@H](C)C)C (-)-INDOLACTAM-V

C1[C@@]2([C@H]([C]3([C@H](C\C=C(\C)C)O3)C)([C@H]([C@@H](OC(NC(CCl)=O)=O)CC2)OC))O1 O-(CHLOROACETYLCARBAMOYL)FUMAGILLOL

C1[CH]2[CH](OC([N]2C(CCl)=O)=O)O[C](*)(*)C1=O BICYCLIC OXAZOLONE DERIVATIVE

C1[CH]2[N](C(O[CH]1[C](*)(*)O[CH]2O)=O)C(CCl)=O BICYCLIC OXAZINONE DERIVATIVE

c12c(c(cc(o1)c1cc(c(N)cc1)F)=O)c(c(F)cc2F)N DIAMINOTRIFLUOROFLAVONES-1

c12c(c3c(cc(O)cc3)O[CH]1c1ccc(OCC[N]3CCCCC3)cc1)sc1c2ccc(c1)O LY326391

c12c(c(c3C(c4cccc(c4C(c3c2O)=O)OC)=O)O)C[C@](C(COC(CCCC)=O)=O)(O)C[C@@H]1O[C@@H]1C[C@@H]([C@H](O)[C@@H](O1)C)NC([C](F)(F)F)=O VALRUBICIN

c12c(cc3ccccc3n2)cccc1C(NCC[N](C)C)=O NSC 601316

O1[C@@H]([n@]2c(nc(N)cc2)=O)[C@H]([C@@H](O)[C@H]1CO[P](OC(CCCCCCCCCCCCCCCCC)=O)(O)=O)O STEAROYL-ARA-CMP

O1[C]2([C@H]1(C=C[C@H]([C@H]1(C[C@@H](C[C@H]3([C@@H](C(O[C@@H](C[C@@H]2OC(CCCCCCCCCCCCCCC)=O)([C@@H]([C@H](\C(=C\C=C/C(=C\c2coc(n2)C)C)C)OC)C))=O)(O3)))(CC(O1)=O)))C))C RS-1541

c1(cnc(=O)[nH]c1)F 5-FLUORO-2(1H)-PYRIMIDINONE

c12c3c4c5C(=O)NCc5c2c2ccccc2[n]1[C]1([C@@H]([C@H]([N@](C(c2ccccc2)=O)C)C[C@H]([n]3c2ccccc42)(O1))OC)C MIDOSTAURIN

c12[C@@H]3([C@@H](CCc1cc(Cl)cc2)([N](C(=O)CC3)C)) LY300502

c1(c2c(ccc1C)nc(N)[nH]c2=O)Sc1ccncc1 AG 337

c12c([C](CC[C]2(C)C)(C)C)cc(C)c(c1)C(c1ccc(C(O)=O)cc1)=C BEXAROTENE

c12[C@]34[CH]5[C]([CH](OC(C)=O)[C@@]([C@@H]3([N](C)c1cc(OC)c(c2)[C@@]1(c2c(c3ccccc3[nH]2)CC[N]2C[C@H](C1)([C@@H]1[C@](C2)(CC)O1))C(OC)=O))(C(OC)=O)O)(C=CC[N]5CC4)CC VINLEUROSINE

c1(c2c(cccc2)[nH]c1)C[C@H](NC([C@@H](NC([C@H](Cc1ccccc1)NC([C@H](Cc1c[nH]cn1)NC([C@@H](NC([C@@H](NC([C@@H](NC([C@H](Cc1ccc(O)cc1)NC([C@@H](NC(C)=O)CO)=O)=O)CO)=O)CCSC)=O)CCC(O)=O)=O)=O)=O)CCCNC(N)=N)=O)C(NCC(N[C@H](C([N]1[C@H](C(N[C@@H]([CH](C)C)C(N)=O)=O)CCC1)=O)CCCCN)=O)=O INTERMEDINE

N([C@H](C(N[C@H](C(N[C@H](C(N[C@H](C(N[C@H](C(N[C@H](C(N[C@H](C(N[C@H](C(N[C@@H](CC(N)=O)C(O)=O)=O)CCC(O)=O)=O)C)=O)CCC(O)=O)=O)CCC(O)=O)=O)[CH](C)C)=O)[CH](C)C)=O)CCC(O)=O)=O)CCCCN)C([C@@H](NC([C@@H](NC([C@@H](NC([C@@H](NC([C@@H](NC([C@@H](NC([C@@H](NC([C@@H](NC([C@@H](NC([C@@H](NC([C@@H](NC([C@@H](NC([C@@H](NC([C@@H](NC([C@@H](NC([C@@H](NC([C@@H](NC([C@@H](NC(C)=O)CO)=O)CC(O)=O)=O)C)=O)C)=O)[CH](C)C)=O)CC(O)=O)=O)[C@@H](C)O)=O)CO)=O)CO)=O)CCC(O)=O)=O)[C@H](CC)C)=O)[C@@H](C)O)=O)[C@@H](C)O)=O)CCCCN)=O)CC(O)=O)=O)C[CH](C)C)=O)CCCCN)=O)CCC(O)=O)=O)CCCCN)=O THYMALFASIN

[198Au] 198 AU GOLD COLLOIDAL

O([C@@H]1O[C@@H]([C@@H](O)[C@@H]([C@H]1O)O)CO)[P](NCCCl)(NCCCl)=O GLUFOSFAMIDE

C1(/[C@H]2([C]([C@@H]([C@@H](\C=C\C=C/[C](CC)(CC)O)C)CC2)(CCC1)C))=C\C=C1\C([C@H](C[C@@H](C1)O)O)=C SEOCALCITOL

C1[CH](CC[N+](C1)(C)C)O[P](OCCCCCCCCCCCCCCCCCC)(=O)[O-]PERIFOSINE

c12c(nc(CNc3ccc(C(N[C@@H](CCC(=O)[O-])C(=O)[O-])=O)cc3)cn1)c(nc(n2)N)N.[Na+].[Na+] AMINOPTERIN SODIUM

c1([N+](=O)[O-])[nH]ccn1 AZOMYCIN

C([N](C=O)O)C(O)=O HADACIDIN

c12c(nc(=O)[n](c1=O)C)[nH]ncn2 REUMITSIN

C([N]1CC1)[CH](C=C)O ETHOXENE TETRAMIN

c1(nc(nc(n1)NCO)NCO)NCO TRIMETHYLOLMELAMINE

C([N](CC(NN)=O)CC(NN)=O)C(NN)=O NITRAZINE

O1[Si]2(OCC[N](CC1)CCO2)CCl CHLOROMETHYLSILATRANE

C([C@H](NC(C)=O)C(O)=O)CC(C=[N+]=[N-])=O DUAZOMYCIN

O1[Si]2(OCC[N](CC1)CCO2)OCC ETHOXYSILATRANE

O([S@@](C)(=O)=O)C[C@H]([C@H]([C@@H]([C@@H](CO[S](C)(=O)=O)O)O)O)O D-MANNITOLBUSULPHAN

O1[C@@H]([n@@]2c([nH]c(=O)c(c2)F)=O)[C@@H]([C@@H](O)[C@H]1CO)O FLUORAFUR

c1([N](CCCl)CCCl)c([nH]c(=O)[nH]c1=O)C CHLORETHYLAMINOURACIL

C([C@@H]([N@@](N=O)O)[CH](C)C)NC(/C=C\C)=O DOPASTIN

c12c(nc[nH]2)ncnc1SCCCCC(O)=O BUTHIOPURINE

c1(c2c(c(ncn2)N)[nH]n1)[C@@H]1O[C@H](CO)[C@H]([C@H]1O)O FORMYCIN

C=1([C@@H](NC(=O)C1C(C)=O)[C@H](CC)C)O TENUAZONIC ACID

N([P]([N]1CC1)([N]1CC1)=O)C(Nc1ncc(OC)cn1)=O EDERPIN

O=[P]([N]1CC1)([N]1CC1)[N]1CCCCCC1 HEXADEPA

O[C@@H]([C@@H]([C@@H](CNCCCl)O)O)[C@@H](CNCCCl)O ZITOFENTON

c12c(C(C=C(C1=O)C)=O)c(ccc2)O PLUMBAGIN

c1(oc([N+](=O)[O-])cc1)/C=C\C=N/[N](CCCl)CCCl NIFURON

N([P]([N]1CC1)([N]1CC1)=O)C(c1ccc(F)cc1)=O FLUORBENZOTEF

c12[n@]([C@@H]3C=C(CO)[C@H]([C@H]3O)O)cnc1c(ncn2)N NEPLANOCIN

O1[C]2([CH]1[CH]([n]1c3c(c(ncn3)N)nc1)[CH]([CH]2O)O)CO NEPLANOCIN C

C=1([C@@H]2([C@@H](C(=CO[C@H]2O)C(OC)=O)(CC1)))CO GENIPIN

O1[C@@]([n@@]2c3c(c(ncn3)N)nc2)([C@@H]([C@@H](O)[C@H]1CO)O)CO PSICOFURANINE

c1([N](CCCl)CCCl)ccc(CC(O)=O)cc1 CHLORPHENACIL

c1([N](CCCl)CCCl)cc(ccc1C)C(O)=O ALKYROM

c12c(N=C[C@H]3([N](C1=O)[CH](CC3)O))cc(O)c(c2)OC NEOTHRAMYCIN

c1([N](CCCl)CCCl)ccc(C[C@H](C(O)=O)N)cc1 MEDFALAN

c1([N](CCCl)CCCl)ccc(CCOC(C)=O)cc1 PHENESTER

c1(c(C[N](CCCl)CCCl)ccc(c1)[N+](=O)[O-])C[CH](C(O)=O)N NITROCAFAN

c1([N](CCCl)CCCl)ccc(CC[CH](C(O)=O)N)cc1 AMINOCHLORAMBUCIL

c12c(oc(c1OC)[CH](C)C)cc1oc(=O)ccc1c2 PEUCEDANIN

c1(/c([nH]\c(=C/[CH](C)C)c([nH]1)=O)=O)=C\c1ccccc1 ALBONOURSIN

c1(C(/C(=C/C(O)=O)Br)=O)ccc(OCCCCC)cc1 PENBEROL

C=1([C]2(CC2)[C](C(=O)C=2C1[C@H]([C@@](CO)(C)C2)O)(C)O)C LAMPTEROL

C1[C@H]2([C@@H]3([C@H](C[N]4[C@@H]2(CCCC4=O))(CCC[N+]3(CC1)[O-])))   OXYMATRINE

C([C@@H]1[C@@H](Cc2cc(ccc2)O)C(=O)OC1)c1cc(ccc1)O   ENTEROLACTONE

O1[C]23[C]1(C(c1cccc(c1[CH]3O)O)=O)[CH]([CH](C)[C]([CH]2O)(C([CH]1[CH](C)O1)=O)O)O
CERVICARCIN

c1([N](CCCl)CCCl)ccc(Oc2ccc(C[CH](C(O)=O)N)cc2)cc1   FENTIRIN

c12c(ccc(c1Cl)CNc1ccc(C(N[C@@H](CC(O)=O)C(O)=O)=O)cc1)nc(N)nc2N   FLUORASQUIN

c12c(cc(CNc3ccc(C(N[C@@H](CC(O)=O)C(O)=O)=O)cc3)cc1)c(nc(n2)N)N   QUINASPAR

c12c(ncc(n1)[CH](Nc1ccc(C(N[C@@H](CCC(O)=O)C(O)=O)=O)cc1)C)nc(N)nc2O   BREMFOL

c12c(ncc(n1)C[N](c1ccc(C(N[C@@H](CCC(O)=O)C(O)=O)=O)cc1)C)nc(N)nc2O   METHOPTERINE

c12c3c(cc(=O)c(cc3)OC)[C@@H](N)CCc2cc(c(c1OC)OC)OC   CITOSTAL

C1([C@@H]2([C@@H](C[C]3(O[C@](C(=C[C@H]2(OC1=O))C)(O)C[C@@H]3O)C)OC(/C(=C/C)C)=O))=C
ANNUITHRIN

C1([N](C(C[N](C1)CC[N]1CC([N](C[N]2CCOCC2)C(C1)=O)=O)=O)C[N]1CCOCC1)=O   BIMOLANE

C1[C@]23[C]4([C@H]([C@@]([C@H](O)CC4)(CO)C)(CC[C@H]2(C[C@H]1([C@](CO)(O)CC3))))C
APHIDICOLIN

c12c(C(=O)c3c2cccc3)cc(OCC[N](C)C)c2c1cccc2   BENFLURON

C1[C@@]23[C@H]4([C]5([C@H](C(=CC([C@H]5O)=O)C)(C[C@H]3(OC([C@@H]([C@H]2([C@@H](C)[C@H]([C]4(O)O1)O))OC(C)=O)=O)))C)   HOLACANTHONE

C([C@@]12[C@H]3([C@]4(C[C@@H](C[C@@H]3O)(C(C4=O)=C))C(=O)O[C@H]1(CC[C]([C@H]2CO)(C)C)))OC(C)=O
RABDOPHYLLIN G

c1([N](CCCl)CCCl)ccc(C[CH](C(N[CH](C(OCC)=O)[CH](C)C)=O)NC(C)=O)cc1   ASALINE

c1(c(c(c(C)c(c1O)Cl)C=O)O)C\C=C(\CC\C=C(\[C@@H]1CC(=O)[C](O1)(C)C)C)C   ASOFURANONE

C1[C@H]2([C@H]3([C@@H](c4c(cc(OC([N](CCCl)CCCl)=O)cc4)CC3)(CC[C]2([C@H](O[P](O)(O)=O)C1)C)))
ESTRAMUSTINE PHOSPHATE

c12c(N[C@H](O)c3[n](C1=O)cc(/C=C\C)c3)c(c(C)c(c2)O[C@H]1[C@H]([C]([C@H](NC)[C@H](O1)C)(C)O)O)O
SIBIROMYCIN

c12[C@]34[C]5([C@H]([N@@](C[CH]6CC6)CC4)Cc2ccc(c1O[C@H]3[C@H]([N@](CCCl)CCCl)CC5)O)O
CHLORNALTREXAMINE

C=1(C(C(=CC(C1)=O)OC)=O)CCCCCCCCC\C=C/CCCCCC   IRISQUINONE A

c12c3c(c(O)c(c1O[C](C)(C)C=C2)C\C=C(/C)C)c(c(c1ccc(O)cc1)co3)=O   OSAIIN

c12c3c(c4c(cc(C)cc4OC)c(o3)=O)cc(c2c(O)ccc1[C@H]1O[C@@H]([C@@H](C)O)([C@@H]([C@H]1O)O))OC
GILVOCARCIN M

c1([N](CCCl)CCCl)ccc(C[C@@H](C(N[C@@H](Cc2ccccc2)C(OCC)=O)=O)NC(C)=O)cc1   ASAFAN

c12c3c([C@@H](NC(C)=O)CCc2cc(c(c1OC)OC)O[C@@H]1O[C@@H]([C@@H](O)[C@@H]([C@H]1O)O)CO)cc(=O)c(cc3)OC   COLCHICOSIDE

C1[C@]2([C]3([C@@]45[C@@H](C=C(C)CC5)(O[C@@H]2(C[C@H]3OC(C=CC=CC(=O)OCC[C@H]([C@@H](C(OC4)=O)O)C)=O)))C)O1   MUCONOMYCIN A

C=1[C@@]23[C@H](c4c(cc5OCOc5c4)CC[N]2CCC3)([C@H](OC([C](CC[C](C)(C)O)(CC(OC)=O)O)=O)C1OC)
HARRINGTONINE

O1[C@]23[C@H]1(C[C@H]1([C]4([C@]([C@@H]([C@@H]([C@H]5(CC(=C(CO)C(O5)=O)C))C)CC4)(C)CC[C@H]1([C]2(C(C=C[C@@H]3O)=O)C))O))   WITHAFERIN A

C1([C@]23[C@H]([C@@H](Cc4ccccc4)N1)([C@@H](C(=C)[C@H]([C@@H]2(C=CC[C@@H](CCC[C@H](C=CC(O3)=O)O)C))O)C))=O CITOCHALASIN B

C1[C@]2([C]3([C@]45[C@@H](C=C(C)CC4)(O[C@@H]2(C[C@H]3OC(C=CC=C[C@H]([C@@H](C)O)(OCC[C@H]([C@@H](C(OC5)=O)O)C))=O)))C)O1 RORIDIN A

C1=2[C@@H]([C@H](C[C@@H]([C@@H]([C@H](C=C([C@H](OC(N)=O)[C@@H](C=CC=C(C(NC(C1=O)=CC(C2)=O)=O)C)C)C)C)OC)OC)C)OC MACBECIN I

c12c3c(c4c(cc(C=C)cc4OC)c(o3)=O)cc(c2c(O)ccc1[C@H]1[C@@H]([C@H]([C@@H](OC(C)=O)[C@@H](O1)C)[N@](C)C)O)OC RAVIDOMYCIN

c12c3c4c(=O)oc1c1c(cccc1O[C@H]1[C@H](O[C@@H]5[C@@H]([C@H]([C@@H](O)[C@H](O5)C)OC)O)[C@H]([C@@H](O)[C@H](O1)C)O)c(c2c(=O)oc3ccc4C)O CHARTREUSIN

c12[C@H]([C@@H]3([C@H](COC3=O)([C@H](c1cc1OCOc1c2)O[C@@H]1O[C@H]2[C@@H](O[CH](c3ccccc3)OC2)[C@@H]([C@H]1O)O)))c1cc(c(OC)c(c1)OC)OC PRORESID

C1=2[C@]3([C@H](C[N]1C(c1cc4c5c([N](C(c6cc7c8c([N](C(N)=O)CC8)c(c(c7[nH]6)OC)O)=O)CC5)c(c(c4[nH]1)OC)O)=O)(C3))c1c([nH]cc1C)C(C2)=O RACHELMYCIN

O1[C@]23[C@H]([C@@H]4([C@](C(C)=C)([C@@H]([C@H]3C)OC(c3ccccc3)=O)O[C]1(\C=C\C=C/CCCCC)O4))([C@H]1([C@@](CO)(O1)[C@H]([C]1([CH]2C=C(C)C1=O)O)O)) GNIDILATIDIN

C1([C@@H]2([C@H](c3ccc(Oc4c(O)ccc(C[C@H]([N]1C)C(N[C@@H](C(N[C@@H](C)C([N]([C@H](C(N[C@H](C([N]2C)=O)C)=O)Cc1ccc(OC)cc1)C)=O)=O)C)=O))c4)cc3)O))=O BOUVARDIN

O([C@H]([C@H](NC(c1nc([C@@H](NC[C@@H](C(N)=O)N)CC(N)=O)nc(c1C)N)=O)C(N[C@@H]([C@H]([C@@H](C(N[C@H](C(NCCc1nc(c2nc(C(NCCCNCCCCN)=O)cs2)cs1)=O)[C@@H](C)O)=O)C)O)C)=O)c1c[nH]cn1)[C@H]1[C@@H](O[C@@H]2[C@H]([C@H]([C@H](O)[C@H](O2)CO)OC(N)=O)O)[C@H]([C@H](O)[C@@H](O1)CO)O BLEOMYCIN A5

O1[C]2([CH]([CH]([CH]3[N](C(C)=O)C3)O)OC(/C(C(C)=O)=N/O)=NC2=O)OC(=O)[CH]([C]1(CNC(c1c2c(cc(c1)OC)c(ccc2)C)=O)C)O CARZINOPHILIN

c12c(oc3c(ccc(c3n1)C(N[C@@H]1C(N[C@@H](C([N]3[C@@H](CCC3)C([N](C)CC([N@]([C@H](C(O[C@@H]1C)=O)[CH](C)C)C)=O)=O)=O)[C@H](CC)C)=O)=O)C)c(c(=O)c(c2C(N[C@@H]1C(N[C@@H](C([N]2[C@@H](CCC2)C([N](C)CC([N@]([C@H](C(O[C@@H]1C)=O)[CH](C)C)C)=O)=O)=O)[C@H](CC)C)=O)=O)N)C ACTINOMYCIN VII

C([C@@H](C(O)=O)N)OCCN OXALYSINE

O1[C@@H]([N@@]2C(NC(N)=NC2)=O)[C@@H]([C@@H](O)[C@H]1CO)O DIHYDROAZACYTIDINE

c1([C@@H]2O[C@H](CO)[C@H]([C@H]2O)O)nc(C(N)=O)c[se]1 SELENAZOFURIN

N([CH]1C(NC(=O)CC1)=O)C(Cc1ccccc1)=O ANTINEOPLASTON A 10

O1[CH]2[CH]3[C]([C]1(C)O)(C(=O)O[CH]3C[CH](C)[CH]1[C]2(C(C=C1)=O)C)C TENULIN

C1[C@@H]2([N]([C@H](C=3C(C(OC)=C(C(C13)=O)C)=O)CNC(C(C)=O)=O)[C@H]([C@H]1(CC3=C([C@H]2([N]1C))C(C(OC)=C(C3=O)C)=O))C#N) SAFRAMYCIN A

c12c(c(c3C(c4cccc(c4C(c3c2O)=O)OC)=O)O)C[C@](\C(=N\N=C2\C[C]([N]([O])[C](C2)(C)C)(C)C)C)(O)C[C@@H]1(O[C@H]1C[C@@H]([C@H](O)[C@@H](O1)C)N) RUBOKSIL-1

C1[C@H]2([C@H]3([C]([C@@H](OC(Cc4ccc([N](CCCl)CCCl)cc4)=O)CC3)(CC[C@@H]2(c2c(cc(OC(Cc3ccc([N](CCCl)CCCl)cc3)=O)cc2)C1))C)) CHLORPHENACYL ESTRADIOL DIESTER

c12c([C@H]([C@](CC)(O)C[C@@H]1O[C@H]1C[C@@H]([C@H](O[C@H]3C[C@@H]([C@H](O[C@H]4C[C@@H]([C@H](O)[C@@H](O4)C)O)[C@@H](O3)C)O)[C@@H](O1)C)[N@@](C)C)C(OC)=O)cc1C(c3c(ccc(c3C(c1c2O)=O)O)O)=O MARCELLOMYCIN

C=1([C](CCCC1C)(C)C)/C=C\C(=C/C=C/C(=C\C(O)=O)C)C ALITRETINOIN

B1234B567B89%10[B]%11%12B%1359[B]47B453[B@@]%12%13[B@@]%115B324(S[Na]SB2457B9%11%12B%13%14%15B%16%17%18[B@@]9%14B94%12[B]4%17[B@@]59[B@@]%184B4%15%16B2%11%13B74)B168B%103 BOROCAPTATE SODIUM B 10

c12c(c(c3ccncc3c2C)C)[nH]c2c1cccc2 ELLIPTICINE

C1[C@@]2([C]3([C]4([C@@H](C=C(C)CC4)(O[C@@H]2(C[C@H]3OC(C)=O)))C)C)O1 TRICHODERMIN

C1[C@](C[C@@H](C)C([C@@H]1[C@@H](C[CH]1CC(NC(C1)=O)=O)O)=O)(OC(C)=O)C ACETOXYCYCLOHEXIMIDE

c12c(c3c(cccc3)cc2)cc(N)c2c1cccc2 CHRYSENEX

N1[P@]([N@@](CCCl)CCCl)(OCC[C@H]1SCC[S](O)(=O)=O)=O.C1[CH](CCCC1)N ASTA Z 7557

O1[C@@H]([C@H](O[CH]1[CH](C)C)CN)CN.C1C(O[Pt]OC1=O)=O SKI-2053R

O1[C@@]23[C]1(CC[C@@H]1([C@@H]([C@H]2(C(C)=CC3))(OC(=O)C1=C)))C ARGLABIN

C1([N]2[C@@H](CC(=C2)/C=C\C(N)=O)([C@H](Nc2c1ccc(c2O)C)C))=O ANTHRAMYCIN

C([C@@H]([C@H](CSc1cccs1)C(NO)=O)C[CH](C)C)(N[C@H](Cc1ccccc1)C(NC)=O)=O BATIMASTAT

c1(c2c(c(/C=N\NC=3NCCN3)c3c1cccc3)cccc2)/C=N\NC=1NCCN1 BISANTRENE

O([C@H]([C@H](NC(c1nc([C@@H](NC[C@@H](C(N)=O)N)CC(N)=O)nc(c1C)N)=O)C(N[C@@H]([C@H]([C@H](C(N[C@H](C(NCCc1nc(c2nc(C(NCCC[S+](C)C)=O)cs2)cs1)=O)[C@@H](C)O)=O)C)O)C)=O)c1c[nH]cn1)[C@@H]1[C@@H](O[C@@H]2[C@H]([C@H]([C@H](O)[C@H](O2)CO)OC(N)=O)O)[C@H]([C@H](O)[C@@H](O1)CO)O BLEOMYCIN A2

c1(c2ccccc2)c(c(nc([nH]1)N)=O)Br BROPIRIMINE

C1(/[CH]([CH]2O[CH](C[CH](OC([CH](C[CH]3C[CH]([C]([C](C[CH]4O[CH](C=C[C]2(C)C)C\C(=C\C(OC)=O)C4)(O3)O)(C)C)OC(C)=O)O)=O)[CH](C)O)C1)OC(/C=C\CCCCC)=O)=C/C(OC)=O BRYOSTATIN-1

c12c(c(c3C(c4cccc(c4C(c3c2O)=O)O)=O)O)C[C@](C(C)=O)(O)C[C@@H]1O[C@@H]1C[C@H]([C@@H](O)[C@H](C1)N)C CARUBICIN

c1([nH]c([nH]c(n1)=O)=O)C(O)=O OTERACIL

c12c(cc(OCCOC)c(c2)OCCOC)ncnc1Nc1cc(ccc1)C#C.Cl ERLOTINIB HYDROCHLORIDE

C=1([C]2([C](C(=O)C=3C1C(=C(C)C3)CO)(C)O)CC2)C IROFULVEN

c12[n](c(c(CC)c1CC)C=C1C(=C(C)C(=N1)C=Nc1cc(OCCOCCOCCOC)c(cc1N=CC1=NC(=C2)C(=C1C)CCCO)OCCOCCOCCOC)CCCO)[Gd](OC(C)=O)OC(C)=O MOTEXAFIN GADOLINIUM

c12[n](c(c(CC)c1CC)C=C1C(=C(C)C(=N1)C=Nc1cc(OCCOCCOCCOC)c(cc1N=CC1=NC(=C2)C(=C1C)CCCO)OCCOCCOCCOC)CCCO)[Lu](OC(C)=O)OC(C)=O MOTEXAFIN LUTETIUM

C1(/[C@H]([n@@]2c(nc(N)cc2)=O)O[C@@H](CO)[C@H]1O)=C\F TEZACITABINE

O1[C@H]([n@@]2c(nc(N)cc2)=O)CO[C@@H]1CO TROXACITABINE

C1[N]([C@H]([C](SC1)(C)C)C(NO)=O)[S](c1ccc(Oc2ccncc2)cc1)(=O)=O PRINOMASTAT

c12c3c(cc4cc([N+](=O)[O-])ccc4n3)C[n]1c(c1COC([C](c1c2)(CC)O)=O)=O RUBITECAN

O([Pt](OC(C)=O)(Cl)Cl)C(C)=O.C1[CH](CCCC1)N.N SATRAPLATIN

c12[n@@]([C@H]3[C@@H]([C@@H]4O[P](O)(=O)OC[C@@H]4O3)O)c(nc1c(ncn2)N)Cl TOCLADESINE

c12c(c3c4c(CC[C@@H]3N)c(C)c(cc4n2)F)C[n]2c1cc1[C](C(=O)OCc1c2=O)(CC)O EXATECAN

c1(c2c(cccc2)[nH]c1)C[C@H]1NC([C@H](Cc2ccc(O)cc2)NC([C@@H](NC([C@@H](Cc2cc3c(cccc3)cc2)N)=O)CSSC[C@H](NC([C@@H](NC([C@@H](NC1=O)CCCCN)=O)[C@H](C)C)=O)C(N[C@@H]([C@@H](C)O)C(N)=O)=O)=O)=O LANREOTIDE

c12c(ccc(o1)=O)cccc2 COUMARIN

c12c([C@H](C[C](C2)(C(CO)=O)O)O[C@H]2C[C@@H]([C@H](I)[C@@H](O2)C)N)c(c2C(c3c(cccc3C(c2c1O)=O)OC)=O)O 4'-DEOXY-4'-IODO-DOXORUBICIN

[nH]1c(cc[nH]c1=O)=O URACIL

O([C@H]([C@H](NC(c1nc([C@@H](NC[C@@H](C(N)=O)N)CC(N)=O)nc(c1C)N)=O)C(N[C@@H]([C@H]([C@H](C(N[C@H](C(NCCc1nc(c2nc(C(NCCCCNC(N)=N)=O)cs2)cs1)=O)[C@@H](C)O)=O)C)O)C)=O)c1c[nH]cn1)[C@H]1[C@@H](O[C@@H]2[C@H]([C@H]([C@H](O)[C@H](O2)CO)OC(N)=O)O)[C@H]([C@H](O)[C@@H](O1)CO)O BLEOMYCIN B2

C1[C@H]([N](C(C[C@H]([C@@H]([N@@](C([C@@H](NC([C@H]([CH](C)C)[N@@](C)C)=O)[CH](C)C)=O)C)[C@H](CC)C)OC)=O)CC1)([C@@H]([C@H](C(N[C@H](c1nccs1)Cc1ccccc1)=O)C)OC) DOLASTATIN 10

C1=2/C([C](CC(C2NC(OC)=O)=O)(C#CC=CC#C[C@H]1O[C@H]1[C@H](O[C@H]2C[C@H]([C@@H](NCC)CO2)OC)[C@H]([C@H](NO[C@H]2C[C@@H]([C@H](SC(c3c(c(c(O[C@H]4[C@@H]([C@@H]([C@@H](O)[C@@H](O4)C)OC)O)c(c3C)I)OC)OC)=O)[C@H](O2)C)O)[C@H](O1)C)O)O)=C\CSSSC CALICHEAMICIN GAMMA1

c12c(oc(c3ccc(O)cc3)cc1=O)cc(O)cc2O APIGENIN

c12[n@@]([C@@H]3O[C@H](CO)[C@H]([C@@H]3O)O)cnc1c(nc(n2)N)OC NELZARABINE

C([CH]1CCC(C)=CC1)(C)=C LIMONENE

C1=2[C@H]([C@H]3([C@]([C@@](\C=C/CO)(O)CC3)(C)C[C@@H]1c1ccc([N](C)C)cc1))(CCC=1C2CCC(C1)=O) LILOPRISTONE

C1[N]([P@@](OCC1)(NCCCl)=O)CCCl (R)-IFOSFAMIDE

C1[N]([P@](OCC1)(NCCCl)=O)CCCl (S)-IFOSFAMIDE

c1([S](Nc2cc(c(OC)cc2)F)(=O)=O)c(c(F)c(c1F)F)F T 138067

C1[C@]2([C@H]3([C@@H]([C]4([C](C(=C(C)[C@H](C4)OC([C@@H]([C@H](c4ccccc4)NC(c4ccccc4)=O)O)=O)[C@H](C([C]3([C@@H](O)C[C@@H]2O1)C)=O)OC(C)=O)(C)C)O)OC(c1ccccc1)=O))OC(C)=O PACLITAXEL

c1c(c2cnccc2)nc(Nc2cc(ccc2C)NC(c2ccc(cc2)C[N]2CC[N](CC2)C)=O)nc1.O[S](=O)OC MCMC00010877

c1([n](c2ccc(cc2c(c1)c1cc(ccc1)Cl)[C](c1cnc[n]1C)(c1ccc(cc1)Cl)N)C)=O MCMC00010884

C=1([C@@H](Oc2c(C1C)ccc(c2)O)c1ccc(cc1)OCC[N]1CCCCC1)c1ccc(cc1)O MCMC00010893

c1([n](c2C(C=C(C(c2c1CO)=O)[N]1CC1)=O)C)/C=C\CO MCMC00010895

C1Cc2c([C@@H](c3c1cc(cc3Br)Cl)[C@@H]1CC[N](CC1)C(C[CH]1CC[N](CC1)C(N)=O)=O)ncc(c2)Br MCMC00010927

C(=O)(CCC(=O)CN)OC.Cl MCMC00010929

c1c2C(/C(=O)Nc2ccc1)=C/c1[nH]c(cc1C)C MCMC00010936

C(C(=O)c1ccc(c2ccc(cc2)Cl)cc1)[C@@H](C(O)=O)CSc1ccccc1 MCMC00010939
